# Continuous invariant-based asymmetries of periodic crystals quantify deviations from higher symmetry

**DOI:** 10.1107/S2052252526002988

**Published:** 2026-06-01

**Authors:** Surya Majumder, Daniel Widdowson, Yury Elkin, Olga Anosova, Andrew I. Cooper, Graeme M. Day, Vitaliy A. Kurlin

**Affiliations:** a Computer Science Department University of Liverpool LiverpoolL69 3BX United Kingdom; b Materials Innovation Factory University of Liverpool LiverpoolL7 3NY United Kingdom; c National Institute for Theory and Mathematics in Biology Chicago USA; d School of Chemistry and Chemical Engineering University of Southampton SouthamptonSO17 1NX United Kingdom; IRCP Chimie-ParisTech, France

**Keywords:** nanocrystals, molecular crystals, structure prediction, nanostructure

## Abstract

Ideal symmetry is known to break down under almost any noise. One measure of asymmetry in a periodic crystal is the relative multiplicity *Z*′ of geometrically non-equivalent units. However, *Z*′ discontinuously changes under almost any displacement of atoms, which can arbitrarily scale up a primitive cell. This discontinuity was recently resolved by a hierarchy of invariant descriptors that continuously change under all small perturbations. We introduce a Continuous Invariant-based Asymmetry (CIA) to quantify (in physically meaningful ångstroms) the deviation of a periodic crystal from a higher-symmetry form. Our experiments on several crystal structure prediction datasets show that about a half of simulated crystals have high values of CIA, while all experimental structures in these datasets have CIA = 0. On another hand, many crystals with high values of *Z*′ in the Cambridge Structural Database (CSD) turned out to be close to more symmetric forms with *Z*′ ≤ 1 due to low values of CIAs.

## Abstract 2

New continuous invariant-based asymmetries quantify deviations of any periodic crystal from its closest higher-symmetry neighbour where all molecules are geometrically equivalent.

## 1. Introduction: motivations for a new continuous asymmetry of crystals

Many periodic crystals are highly symmetric, because a globally stable structure is usually formed by a few energetically favourable interactions, bonds, molecules or formula units, that are repeated in 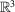 by symmetries (Lax, 2001 ▸). Though we were motivated by molecular crystals, our invariant-based approach to asymmetry extends to all non-molecular crystals and periodic sets in any Euclidean space 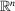.

While molecular crystals can contain many molecules in primitive unit cells, they are often obtained from a smaller number of molecules by *symmetry operations* that are *isometries* (distance-preserving transformations) of 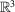 preserving the whole crystal (Chapuis, 2024 ▸). For a non-molecular crystal, the chemical analogue of a molecule is a *formula unit* that is an electronically neutral group of atoms or ions, embedded in 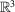 and representing their relative numbers in a given compound, reduced to the smallest integer numbers. For example, table salt has the empirical formula NaCl with a formula unit consisting of two ions, Na^+^ and Cl^−^. This pair of ions can be chosen in many geometrically different ways, because ionic bonds in NaCl do not define a finite bounded object, such as a molecule. Formula units of non-molecular crystals should be single ions, or metal blocks and organic linkers in a metal–organic framework.

In this paper, a *crystal S* means a periodic crystal, while *Z* can be non-integer for disordered or aperiodic crystals (Senechal, 1996 ▸). The *multiplicityZ*(*S*) usually denotes the number of formula units in a primitive unit cell *U* of *S*. If *S* consists of chemically equivalent molecules, the *relative multiplicityZ*′ (*Z prime*) often denotes the number of *symmetry-independent* molecules that can not be matched by symmetries of *S* (Steed & Steed, 2015 ▸). An *asymmetric unit* of *S* is a minimal and simply connected subset 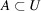, whose images under all symmetry operations of *S* tile the space 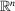. For *co-crystals* with chemically different molecules, van Eijck & Kroon (2000 ▸) used another notation, *Z*′′, for the total number of molecules in an asymmetric unit. To cover non-molecular crystals, we define the relative multiplicity *Z*′(*S*) below for any periodic point set 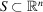 with a motif *M* split into geometric blocks.

**Figure 1:**
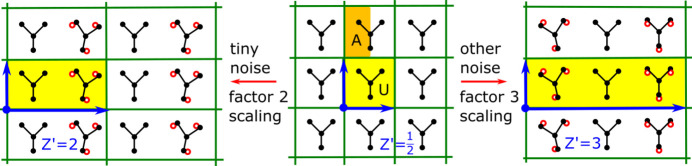
An asymmetric unit *A* in orange can be a half of a unit cell *U* in yellow. Almost any noise can arbitrarily scale up a primitive cell *U* and discontinuously changes the relative multiplicity *Z*′ of a crystal, where molecules are represented by *Y* graphs whose terminal vertices have initial positions shown by red circles.

Definition 1 (a periodic point set S and its relative multiplicityZ′) Any linear basis 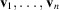 of 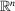 defines the *lattice*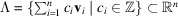 and the primitive *unit cell*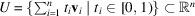. For a finite set of points 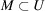 (called a *motif*), a *periodic point set* is 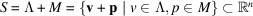. Let an asymmetric unit *A* of *S* overlap with geometric blocks *F*_1_, …, *F*_*g*_, which in the case of a periodic crystal *S* are formula units, such as full molecules, atoms or ions. For *i* = 1, …, *g*, let *F*_*i*_ have *k*_*i*_ symmetry operations (including the identity) that preserve both *S* and *F*_*i*_. Then the *relative multiplicity* is defined as 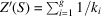, see Fig. 1 ▸.

□

The most general option for any periodic point set 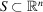 is to split the finite subset *S* ∩ *A* of size (say) *g* into individual points *B*_1_, …, *B*_*g*_. For molecular crystals 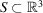, geometric blocks *B*_1_, …, *B*_*g*_ will be connected parts of molecules, as specified in a Crystallographic Information File (CIF). For example, if *S* is a crystal of benzene molecules C_6_H_6_, then one block *B*_*i*_ can be a half-molecule, C_3_H_3_, or one sixth, CH.

Blocks *B*_*i*_, *B*_*j*_ of any 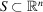 are (isometrically) *equivalent* if there is an isometry of 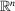 that maps *S* to *S*, and *B*_*i*_ to *B*_*j*_. If all molecules of a crystal 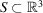 are equivalent, then an asymmetric unit *A* of *S* overlaps with one molecule *F*. In this case, 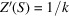, where *k* is the number of symmetry operations preserving both *S* and *F*.

The periodic point set 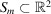 in Fig. 1 ▸ (middle) has one full geometric block *Y* in a unit cell *U*, which is preserved together with *S*_*m*_ by *k* = 2 symmetries, including the reflection across the vertical line that splits *U* into an asymmetric unit *A* and its mirror image, so 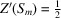. The table salt crystal NaCl has one ion (Na^+^ or Cl^−^) in its asymmetric unit with point group of order 48, so 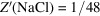.

In about 90% of entries in the Cambridge Structural Database (CSD), an asymmetric unit includes only one molecule, so *Z*′ ≤ 1 (Anderson *et al.*, 2006 ▸). However, the CSD has many crystals with high *Z*′ (Brock, 2016 ▸), *e.g.* OGUROZ has *Z*′ = 56.

Crystal structure prediction (CSP) often starts with simulating *Z*′ = 1 crystals for the most frequent space groups, but a final energy relaxation can produce structures with *Z*′ values up to 36 (Pulido *et al.*, 2017 ▸). More importantly, almost any displacement of atoms or a whole rigid molecule discontinuously changes the size of a primitive (or reduced) cell and hence arbitrarily scales up *Z*′. Fig. 1 ▸ shows nearly identical structures with 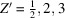 and similarly applies to any periodic crystal.

Ignoring any noise up to a small threshold ɛ only shifts the problem from 0 to another number without guarantees of a continuous change. This *sorites* paradox (when a heap of sand stops being a heap while grains are removed one by one) has been known since ancient times (Wikipedia, 2024 ▸). Its rigorous solution requires an *invariant* that is preserved by any rigid transformation and continuously changes under perturbations of atoms.

While a full hierarchy of invariants for periodic crystals from the computationally fastest to complete is being finalized (Anosova & Kurlin, 2025 ▸; Widdowson & Kurlin, 2025*b* ▸), our continuous asymmetry will be based on the Pointwise Distance Distribution (PDD), which distinguishes all non-duplicate crystals in the world’s largest databases within two hours on a modest desktop computer (Widdowson & Kurlin, 2022 ▸).<!?tpb=12pt>

## 2. Generically complete and continuous isometry invariants of crystals

This section recalls isometry invariants, which will be used to define a continuous invariant-based asymmetry in Section 3. Definition 1[Statement definition1] introducing a periodic crystal *S* in terms of a basis and a motif is widely used for representing crystals in Crystallographic Information Files (CIFs), but is highly ambiguous in the sense that infinitely many pairs (basis, motif) represent the same crystal *S*. This ambiguity motivated us to distinguish between a crystal *S* and its *structure*, defined as the equivalence class of all periodic sets of atoms that are represented by different CIFs but can be exactly matched with each other by rigid motion, see Definition 6 in Anosova *et al.* (2024 ▸).

Any canonical (standard or conventional) choice of a cell for a periodic crystal is discontinuous under almost any noise, as in Fig. 1 ▸, which was experimentally demonstrated already in 1965 (see p. 80 in Lawton & Jacobson, 1965 ▸). The new definition of a *crystal structure* as a rigid class (consisting of all crystals that can be exactly matched under rigid motion) has become practical due to the hierarchy of invariants that uniquely identify any crystal structure independent of its initial representation.

Definition 2[Statement definition2] introduces the invariant PDD for any periodic set of points in 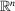, which can be all atomic centres of a crystal in 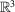, or other points defined by a crystal, for example, atoms of one specific type, or molecular centres, which form a periodic set.

Definition 2 (Pointwise Distance Distribution PDD) Let 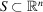 be a periodic point set with a motif *M* = {*p*_1_, *p*_2_, …, *p*_*m*_}. Fix an integer *k* ≥ 1. For every *p*_*i*_ ∈ *M*, let 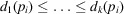 be the distances from *p*_*i*_ to its *k* nearest neighbours within the full set *S*, not restricted to any cell. The matrix *D*(*S*; *k*) has *m* rows consisting of the distances *d*_1_(*p*_*i*_), …, *d*_*k*_(*p*_*i*_) for *i* = 1, …, *m*. If any *l* ≥ 1 rows are identical to each other, we collapse them into a single row and assign the weight *l*/*m* to this row. The resulting matrix of *k* columns and at most *m* rows, complemented by an extra (say 0th) column listing the weights, is the *Pointwise Distance Distribution* PDD(*S*; *k*).

□

The columns of the matrix PDD(*S*; *k*) are ordered because each row consists of increasing values of distances to neighbours but without their indices. So PDD(*S*; *k*) importantly differs from the matrix of pairwise distances between *m* points in the motif *M*, also because neighbours are not restricted to any (extended) cell of *S*.

Since many crystals consist of indistinguishable atoms, we consider all points of *S* unordered. Then PDD(*S*; *k*) has unordered rows and can be interpreted as a discrete distribution of rows (or unordered points in 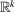) with probabilities equal to the weights assigned to rows. The Pair Distribution Function is obtained from a single collection of all interatomic distances (usually normalized by frequencies and then smoothed) and hence is naturally weaker than PDD(*S*; *k*), which splits distances per point and avoids losing information under smoothing, see the discussion at the end of Section 3 in Widdowson & Kurlin (2022 ▸). This probabilistic interpretation allows one to compare PDDs by many distance metrics on discrete distributions. We usually use the simplest metric called Earth Mover’s Distance (EMD), which was adapted for comparing chemical compositions (Hargreaves *et al.*, 2020 ▸). Theorem 4.2 in Widdowson & Kurlin (2026 ▸) proved that PDD(*S*; *k*) continuously changes in EMD under perturbations, including those that arbitrarily scale up a minimal cell as in Fig. 1 ▸.

The most important strength of the PDD is its generic completeness: Theorem 5.8 in Widdowson & Kurlin (2026 ▸) proved that PDD(*S*; *k*) with a lattice of *S* and the number *m* of points in a motif of *S* suffice to reconstruct any generic periodic point set 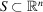, uniquely under isometry, for a large enough *k* with an explicit upper bound. In other words, PDD(*S*; *k*) with a few extra invariants provably distinguishes all crystals, possibly except singular examples that form a subspace of measure 0 within the continuous space of all periodic crystals. In practice, PDD(*S*; *k*) distinguished all non-duplicate crystals in the world’s major databases within two hours on a modest desktop computer, see Table 3 in Widdowson & Kurlin (2026 ▸). Theorem 3.7 in Widdowson & Kurlin (2026 ▸) proved that, as *k* → +∞, the distances in each row of PDD(*S*; *k*) asymptotically approach 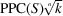, where the Point Packing Coefficient PPC(*S*) is inversely proportional to the point density, as defined below. This fact motivated us to subtract this asymptotic curve from PDD(*S*; *k*) to neutralize the influence of density.

Definition 3 (invariants PPC(S) and PDA(S; k)) Let 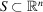 be a periodic set with *m* points in a unit cell *U* of *S*. The *Point Packing Coefficient* is 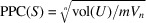, where vol(*U*) is the volume of *U*, and *V*_*n*_ is the volume of the unit ball in 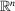. The *Pointwise Deviation from Asymptotic* is the matrix PDA(*S*; *k*) obtained from PDD(*S*; *k*) by subtracting 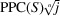 from each distance in columns *j* = 1, …, *k*.

□

Another advantage of PDA(*S*; *k*) versus the original PDD(*S*; *k*) is the experimental convergence to 0 of the *k*th values from the last column of PDA(*S*; *k*) as *k* → +∞, see Fig. 4 in Widdowson & Kurlin (2025*a* ▸). This convergence to 0 was formally proved for any cubic lattice 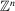 with *n* ≥ 2, see Example SM3.1 in Widdowson & Kurlin (2026 ▸).

Hence, there is no need to substantially increase the number *k* of neighbours, because more distant neighbours bring smaller contributions. We consider *k* not as a parameter that seriously affects PDA(*S*; *k*), but as a degree of approximation like the number of decimal places on a calculator. Column averages of PDA(*S*; *k*) for *k* = 100 suffice to distinguish all non-duplicate crystals in the CSD (Widdowson & Kurlin, 2024 ▸) and can be used as analytic coordinates on geographic-style maps of any materials database, first done for 2D lattices in Bright *et al.* (2023*b* ▸), Bright *et al.* (2023*a* ▸) and Kurlin (2024 ▸).

## 3. A continuous invariant-based asymmetry (CIA) of periodic crystals

The discontinuity of *Z*′ from Definition 1[Statement definition1] under almost any perturbation has been known for over 30 years. The quote ‘two fairly unsymmetrical objects can be combined into a less unsymmetrical structural dimer’ from Wilson (1993 ▸) means that a crystal with *Z*′ = 2 can be geometrically close to a more symmetric crystal with *Z*′ = 1.

This section first defines the Earth Mover’s Distance (EMD) between geometric blocks within a periodic point set 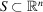 by using the isometry invariant PDA(*S*; *k*) from Definition 3[Statement definition3]. The continuous invariant-based asymmetry of *S* will be defined through EMDs between all blocks in an asymmetric unit of *S*. The EMD needs a ground metric between vectors 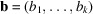 and 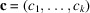 in 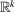, which can be rows of PDA(*S*; *k*). The simplest choices are the *Chebyshev*metric 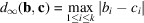 and the *Root Mean Square* (RMS) 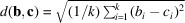.

These ground metrics respect the continuity under perturbations as follows. If any *b*_*i*_, *c*_*i*_ are perturbed up to ɛ, then |*b*_*i*_ − *c*_*i*_| ≤ 2ɛ for *i* = 1, …, *k*, and both 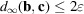, 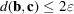. We usually write *d* without a subscript for brevity. If *d*_∞_ is used in computations, all relevant distances and asymmetry will have the subscript ∞.

For any periodic set in 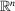, Definition 4[Statement definition4] introduces a distance between geometric blocks *B*, *C* (considered as finite sets of points), which can represent molecules, ions or other well defined disjoint subsets for crystals in 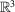. This distance measures how the positions of *B*, *C* differ within a common periodic set *S* containing both *B*, *C*. If *B*, *C* can be exactly matched by isometry of 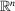 preserving *S*, then this distance is 0. In real examples, any deviation from symmetry should be positive due to noise.

Though the EMD makes sense for distributions of different sizes, our experiments on real crystals will use the EMD only for geometric blocks that are chemically identical. In general, we assume that every point in a periodic set 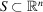 has a categorical label, which is an analogue of an atomic type, such as Na^+^ and Cl^−^.

Briefly, the EMD optimally splits and transports objects from one distribution to another by minimizing the overall cost based on a ground distance between objects. If we need to guarantee matching of points only with the same label (atomic types in a crystal), the ground distance can be adjusted by taking the maximum of *d*_∞_ or *d* = RMS with a discrete metric that is infinite between points of different labels.

Definition 4 (Earth Mover's Distance EMD between geometric blocks) Let 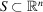 be a periodic set of labelled points with an asymmetric unit *A*. Let 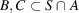 be geometric blocks (finite sets) that have *m*(*B*), *m*(*C*) points of weights 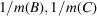, respectively. For a fixed *k* ≥ 1, *i* = 1, …, *m*(*B*), and *j* = 1, …, *m*(*C*), let *R*_*i*_(*B*), *R*_*j*_(*C*) be the rows of *i*th and *j*th points of *B*, *C*, respectively, in the matrix PDA(*S*; *k*) from Definition 3[Statement definition3]. The *Earth Mover’s Distance* is 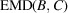 = 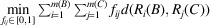, where the minimum is over all variable parameters *f*_*ij*_ ∈ [0, 1] subject to the conditions 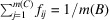 for any fixed index *i* = 1, …, *m*(*B*), and 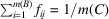 for any fixed index *j* = 1, …, *m*(*C*).

□

The distance EMD(*B*, *C*) measures the minimum perturbation of the rows of the geometric blocks *B*, *C* in PDA(*S*; *k*) to match (distance-based invariants of) *B* and *C* within the ambient periodic set *S*. This perturbation matching *B* and *C* reduces the number of isometrically non-equivalent blocks and hence makes *S* more symmetric.

If an asymmetric unit *A* of *S* has only one geometric block *B*, then *S* has no asymmetry because all blocks in *S* are images of *B* under symmetry operations of *S*. If *A* has only two blocks *B* and *C*, then EMD(*B*, *C*) can be considered an asymmetry of *S*. Definition 5[Statement definition5] introduces the continuous asymmetry in the most general case.

Definition 5 (Continuous Invariant-based Asymmetry CIA(S)) Let a periodic set 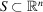 with labelled points have an asymmetric unit *A* consisting of geometric blocks *B*_1_, …, *B*_*g*_. We represent each block *B*_*i*_ by an unordered distribution of rows of adjusted distances in PDA(*S*; *k*) from Definition 3[Statement definition3] for all *p* ∈ *B*_*i*_. Then EMD(*B*_*i*_, *B*_*j*_) denotes the Earth Mover’s Distance between these distributions in Definition 4[Statement definition4]. For any fixed *i* = 1, …, *g*, set 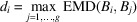. The *Continuous Invariant-based Asymmetry* is 
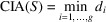
. The *average* version is 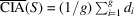.

□

Lemma 7[Statement lemma7] will prove that CIA(*S*) is independent of an asymmetric unit of *S*.

Example 6 (CIAs of periodic sequences in {\bb R}) The periodic sequence 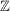 of integers has the motif 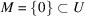 in the primitive cell *U* = [0, 1), which coincides with an asymmetric unit *A*. The only 1-point block 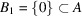 is preserved together with 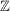 by two symmetry operations (identity and *p* → −*p*), so 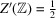. For *k* = 4, the point 0 ∈ *M* has four neighbours  ± 1,  ± 2 in 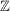 at distances 1, 1, 2, 2, respectively. In Definition 2[Statement definition2], 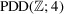 is the single row (1; 1, 1, 2, 2), where the first (0th) entry is weight 1 of 0 ∈ *M*. In Definition 3[Statement definition3], we have *V*_1_ = 2, *m* = 1, vol(*U*) = 1, so 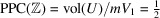. Then 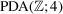 is the single row 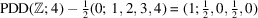. Since 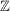 has an asymmetric unit of one point (block), 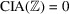 by Definition 5[Statement definition5].

For any small ɛ > 0, consider the perturbed periodic sequence 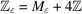 with the larger motif *M*_ɛ_ = {0, 1, 2 + ɛ, 3 + ɛ} and primitive cell *U*′ = [0, 4). Each point *p* ∈ *M*_ɛ_ has only one symmetry operation (identity) that preserves both *p* and 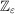, so 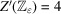 is very different from 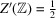. Since the size |*M*_ɛ_| equals vol(*U*′) = 4, we get 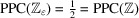. The four points *p* ∈ *M*_ɛ_ have distances to four nearest neighbours in 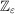 listed below for *k* = 4 in the PDD rows: 
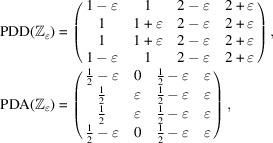
where we skipped the first (0th) column containing equal weights 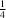 of points. The coincidences of rows 1,4 and rows 2,3 is explained by the symmetry 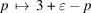 of 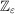. If all four points *p*_*i*_ ∈ *M*_ɛ_ are considered as individual blocks, they are represented by the corresponding rows in 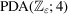. The Earth Mover’s Distances EMD(*p*_*i*_, *p*_*j*_) for *i* ≠ *j* coincide with the 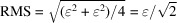 between these points (PDD rows). Then 
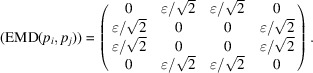
By Definition 5[Statement definition5], for any *i* = 1, 2, 3, 4, we get 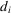 = 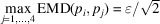. Hence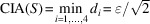 and 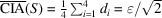. If instead of 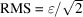, we use the Chebyshev metric *L*_∞_ = ɛ between non-equal PDD rows, we similarly get 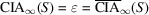.

For 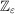, if we choose an asymmetric unit 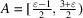 of length 2, which contains only points *p*_1_ = 0 and *p*_2_ = 1, we get the same CIAs by using distance 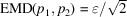 or EMD_∞_(*p*_1_, *p*_2_) = ɛ instead of the 4 × 4 matrix of EMD(*p*_*i*_, *p*_*j*_) above.

If we consider pairs *B*_1_ = {0, 1} and *B*_2_ = {2 + ɛ, 3 + ɛ} within *M*_ɛ_ as geometric blocks, the asymmetric unit *A* contains only *B*_1_. This splitting into blocks gives 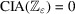, because *B*_2_ is obtained from *B*_1_ by the symmetry 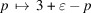. Hence 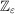 has a higher symmetry at this block level than at the point level.

□

In general, the *g* × *g* matrix of distances (EMD(*B*_*i*_, *B*_*j*_)) describes the relative positions of *g* blocks within an asymmetric unit of *S* in terms of their distances to atomic neighbours within the full *S*. For *i* = 1, …, *g*, the distance *d*_*i*_ measures how far *B*_*i*_ is from all other blocks. The standard (min-max) formula of CIA(*S*) means that the optimal *i*th block *B*_*i*_ serves as a centre minimizing its distance EMD(*B*_*i*_, *B*_*j*_) to the farthest block *B*_*j*_, while 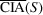 averages maximum deviations *d*_*i*_ from all blocks considered as centres. The default notation CIA(*S*) uses EMD based on the ground distance *d* = RMS between rows of PDA(*S*; *k*) with *k* = 100. For the Chebyshev distance *d*_∞_, we keep the subscript ∞ in the notations EMD_∞_, CIA_∞_ and 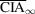.

For all versions of CIA(*S*), the zero value implies that all geometric blocks are isometrically equivalent (transitive under the action of the symmetry group of *S*), *i.e.* any blocks *B*_*i*_, *B*_*j*_ can be exactly matched by an isometry of 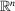 that maps *S* to itself.

Lemma 7 (invariance of CIAs) All CIAs in Definition 5[Statement definition5] are invariant under isometry and independent of an asymmetric unit *A* of any periodic point set 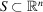.

□

Lemma 7[Statement lemma7] and all further results below are proved in Appendix B[App appb].

Lemma 8 (inequalities for CIAs) In the notations of Definition 5[Statement definition5], CIA ≤ CIA_∞_, 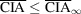 and 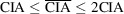 hold for any periodic point set 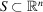.

□

Since Definition 5[Statement definition5] is based on the invariant PDA(*S*; *k*), the full notation should be CIA(PDA(*S*; *k*)), where PDA(*S*; *k*) can be replaced with another ‘pointwise’ invariant, such as the higher-order PDA^(*h*)^ (Widdowson & Kurlin, 2025*b* ▸) or complete isoset (Anosova *et al.*, 2026 ▸). In this paper, we use only PDA(*S*; 100) and write CIA(*S*) for brevity. Theorem 9[Statement theorem9] justifies the continuity of the asymmetry CIA(*S*) under small perturbations of points, including those that arbitrarily scale up a primitive cell of *S*.

Theorem 9 (continuity of CIA under perturbations) Let 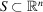 be a periodic point set and *r*(*S*) denote the minimum half-distance between any points of *S*. For any 0 < ɛ < *r*(*S*), let a periodic point set 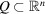 be obtained by perturbing every point of *S* up to Euclidean distance ɛ. Then all versions of CIAs in Definition 5[Statement definition5] based on the invariant PDA(*S*; *k*) for any *k* ≥ 1 satisfy |CIA(*S*) − CIA(*Q*)| ≤ 4ɛ.

□

For a periodic point set 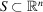 with a motif of *m* points, the invariant PDD(*S*; *k*) based on *k* atomic neighbours can be computed in asymptotic time 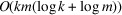, which is near-linear in both *m*, *k*, see details in Theorem 3.10 in Widdowson & Kurlin (2026 ▸). Theorem 10[Statement theorem10] estimates extra time for computing CIAs in Definition 5[Statement definition5].

Theorem 10 (computational complexity of CIA) Let a periodic point set 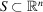 have an asymmetric unit *A* of *g* blocks *B*_1_, …, *B*_*g*_, each consisting of at most *m* points, respectively. Starting with PDD(*S*; *k*), all versions of CIAs can be computed in time 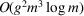. If *A* consists of *m* single-point blocks, then the time is *O*(*m*^3^).

□

The polynomial-time complexity in Theorem 10[Statement theorem10] speeds up another approach to molecular asymmetries via the Continuous Similarity Measure, which is minimized over exponentially many permutations of given atoms (Tuvi-Arad & Alon, 2026 ▸).

## 4. Fast detection of asymmetric crystals in large simulated CSP datasets

This section visualizes several versions of CIA for over fifty thousand simulated crystals from four CSP datasets reported in Pulido *et al.* (2017 ▸). At that time, the synthesized crystals predicted by these CSPs substantially extended the small population of nanoporous crystals in the CSD. However, these predictions took more than 12 weeks on a supercomputer, also due to predictions of properties, such as gas capture.

**Figure 2:**
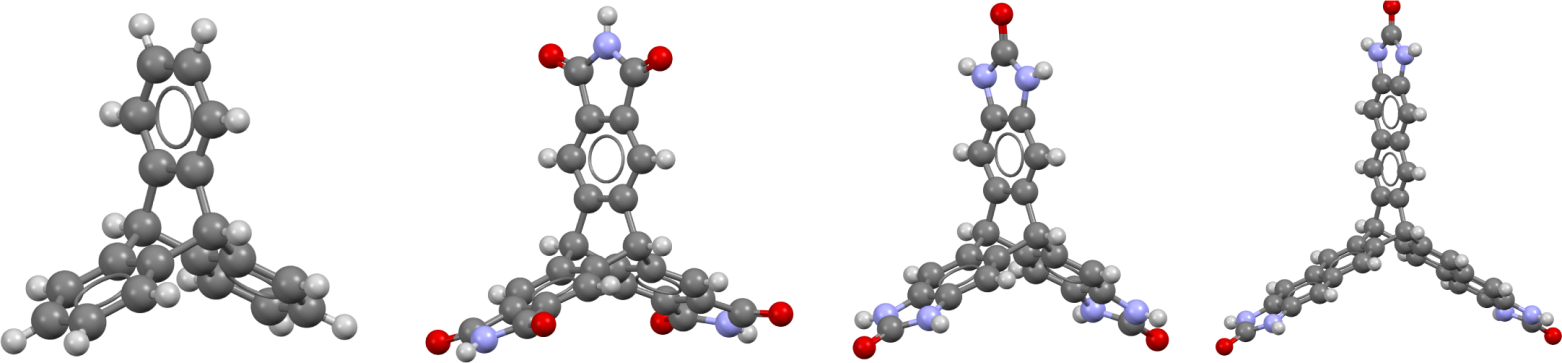
From left to right: T0, T1, T2 and T2E molecules in the four CSP datasets in Section 4.

In these cases, all experimental crystals have an asymmetric unit consisting of a single molecule, hence CIA = 0 for all versions, which confirms the symmetry principle saying that real crystals tend to be highly symmetric. All simulated crystals in the four CSP datasets are based on one of the four molecules in Fig. 2 ▸.

Since each molecule has a rigid shape of three symmetric ‘arms’, its position in 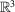 is uniquely determined by three base points at the ends of these ‘arms’, selected as follows. T0: mid-points defined by three pairs of the most distant carbons from the centre. T1: three nitrogens. T2 and T2E: three oxygens. All values of CIAs in this section were computed on periodic sets obtained by replacing each molecule with its three base points. The alternative option of considering all atoms is slower and unnecessary in these cases, because three base points per molecule suffice to completely reconstruct every crystal based on one of the molecules T0, T1, T2 and T2E in Fig. 2 ▸.

**Figure 3:**
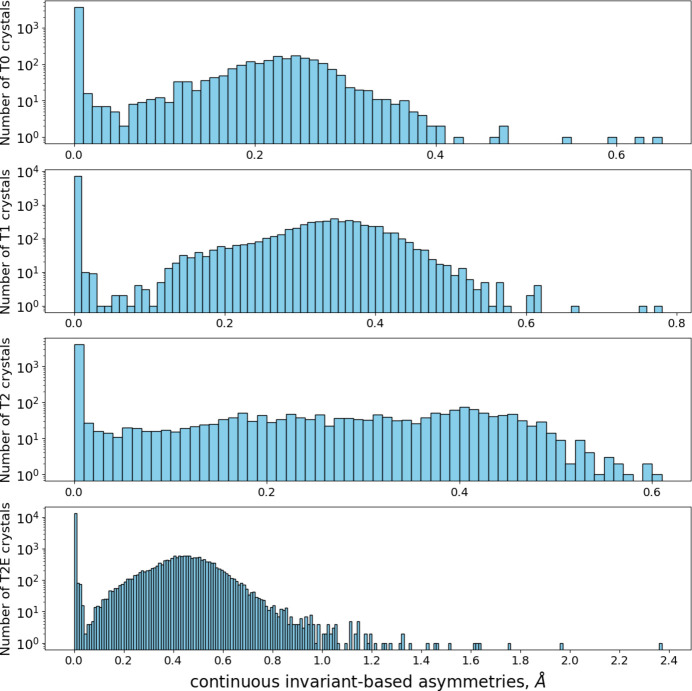
The histograms of CIA for simulated crystals represented by three base points at ‘ends’ of molecules in Fig. 2. Row 1: T0. Row 2: T1. Row 3: T2. Row 4: T2E.

Fig. 3 ▸ has four histograms of the default CIA across four CSP datasets. In each histogram, the vertical *y* axis shows the number of crystal structures on the log scale (as powers of 10) whose CIAs fall in a bin of size 0.01 Å. The first vertical bin with CIA = 0 represents all crystals with CIA = 0. Since any CIA in Definition 5[Statement definition5] is a min-max or an average of non-negative distances, all versions of CIAs vanish simultaneously.

All structures in the four CSP datasets were generated with *Z*′ = 1. The last stage of energy minimization allowed this symmetry to be broken, which explains many cases of *Z*′ > 1 in Table 1 ▸. If the generation stage included structures with *Z*′ ≥ 2, optimized crystals might have different distribution of CIAs than in Fig. 3 ▸.

In Appendix A[App appa], Fig. 20 contains histograms of CIA_∞_ based on EMD_∞_ with the ground metric *d*_∞_ in Definition 4[Statement definition4]. The Chebyshev metric *d*_∞_ captures the largest deviations, while *d* = RMS averages over *k* = 100 adjusted interatomic distances, hence CIA_∞_ has a larger range in comparison with CIA, see the maximum values in Table 1 ▸.

**Figure 4:**
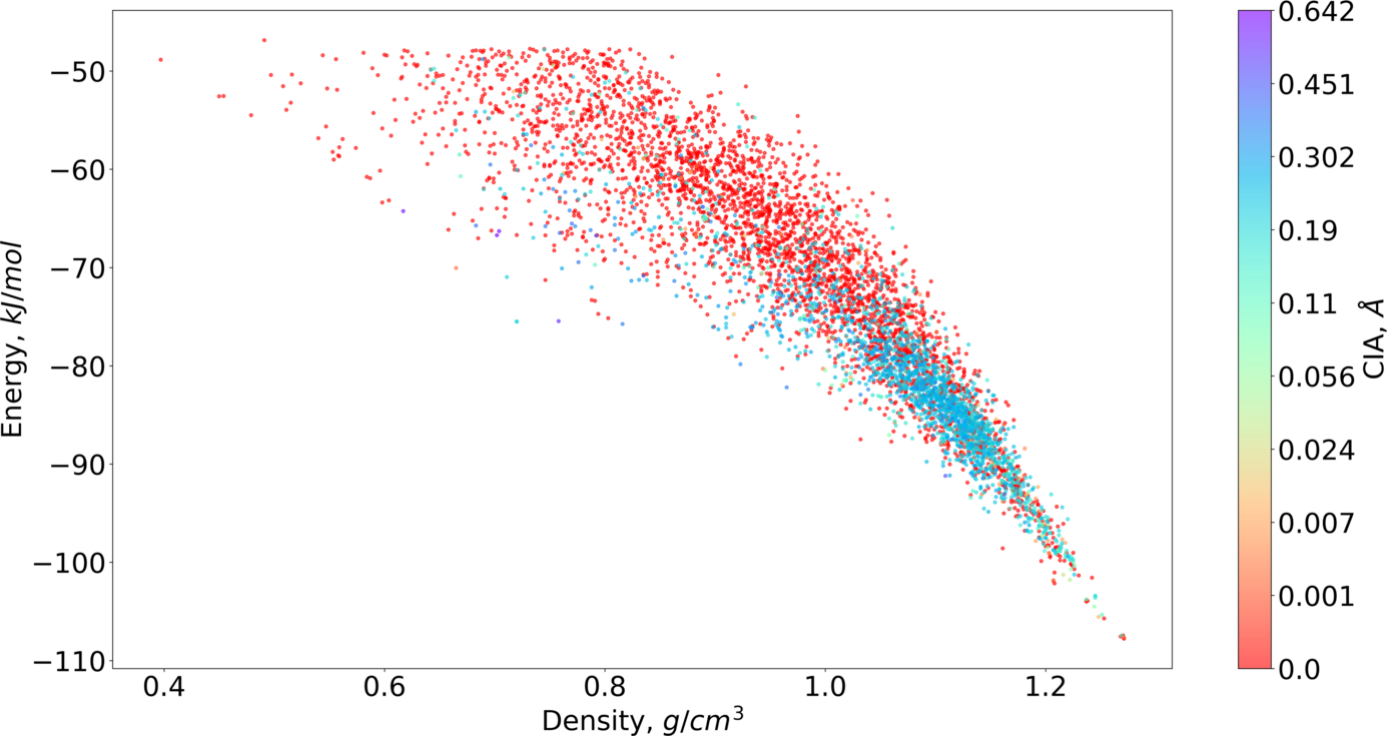
Energy versus density for simulated T0 crystals, coloured by their CIA.

CSP datasets are often visualized via energy–density plots, because density is a fast and continuous invariant. Moreover, density usually indicates stability, because real crystals tend to be dense. Figs. 4 ▸, 5 ▸, 6 ▸ and 7 ▸ show these energy–density plots, where each crystal is represented by a point (density, energy), coloured according to its CIA. The colour bars on the right-hand side of the plots show the CIA range, with the bright red colour corresponding to high-symmetry structures with CIA = 0.

Table 1 ▸ highlights that large subsets (between 30% and 55%) of each CSP dataset have CIA > 0. Since all experimental crystals based on these molecules have CIA = 0, all non-symmetric crystals with CIA > 0 are likely non-ideal approximations to symmetric synthesized crystals. Indeed, if all non-red dots are removed from Figs. 4 ▸, 5 ▸, 6 ▸ and 7 ▸, the remaining red dots will still form roughly similar landscapes with all ‘minimal spikes’ of density represented by only symmetric crystals with CIA = 0 in red.

The Pearson correlation *r*(energy, density) in Table 1 ▸ reflects the inverse dependence on density, because denser crystals tend to be more stable and have lower energies. This inverse correlation is the strongest with *r* = −0.909 for crystals based on the smaller molecule T0 and is still noticeable for crystals based on the larger molecules T1, T2 and T2E. The new asymmetry CIA is linearly independent of density and energy due to its low correlations, especially for the T2 and T2E datasets. All experimental crystals based on these molecules have CIA = 0, but their closest simulated analogues may not have the lowest energies as for the nanoporous polymorph T2-γ.

**Figure 8:**
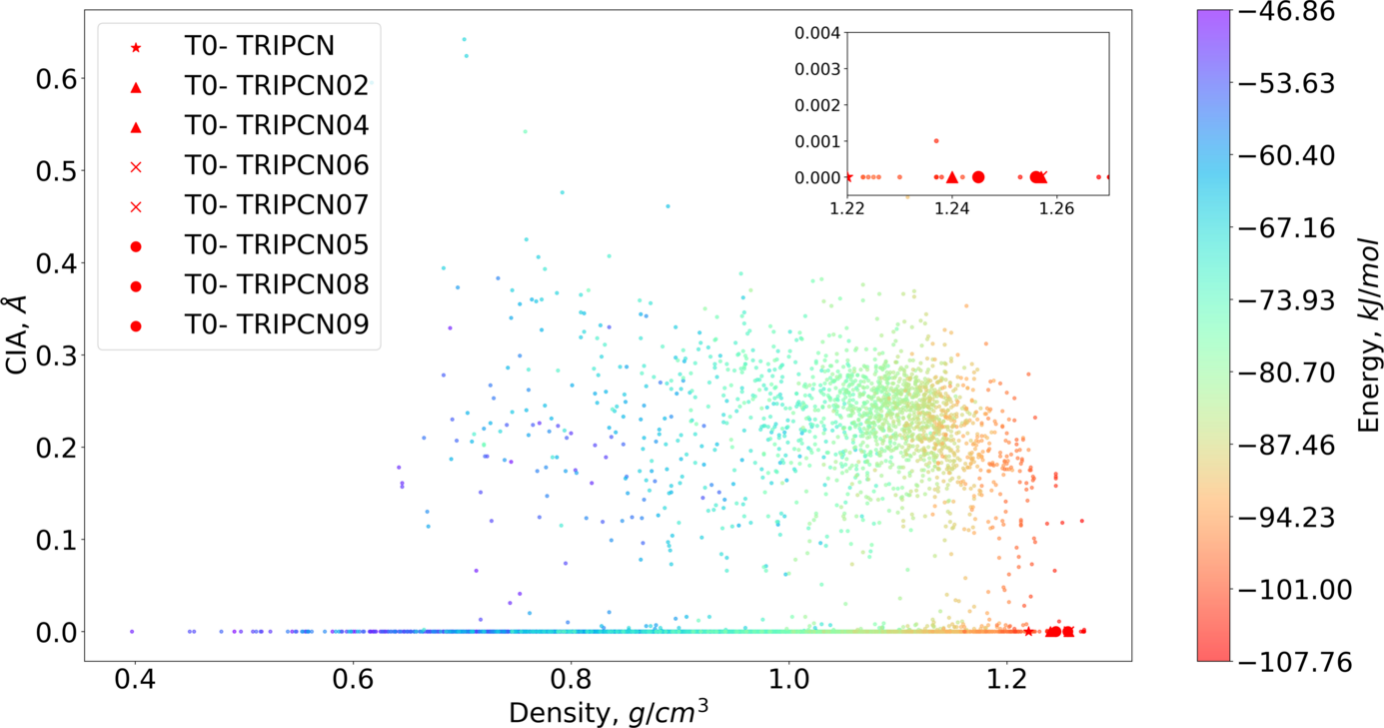
CIA versus density for simulated and experimental T0 crystals in the CSD.

Figs. 8 ▸, 9 ▸, 10 ▸ and 11 ▸ show experimental crystals by red marks of various shapes in the coordinates (density, CIA) and indicate their apparent independence. In each figure, the top right corner includes a zoomed-in image containing experimental crystals that are closest by density. While many simulated crystals are symmetric with CIA = 0, all non-symmetric crystals form noisy clouds with energies across full colour bars.

The visible gaps between these clouds and the horizontal axis CIA = 0 confirm a local version of the symmetry principle saying that a nearly symmetric simulated structure likely converges to a higher symmetry structure with CIA = 0.

**Figure 12:**
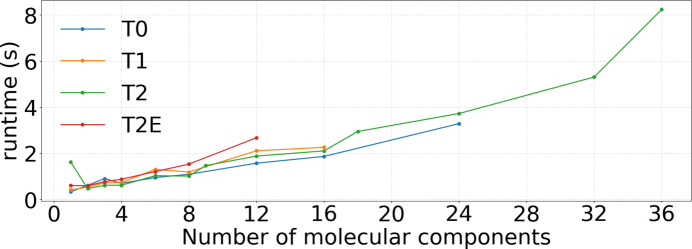
Average running times (in seconds) of CIA calculations on four CSP datasets versus the number *g* of molecules in asymmetric units, performed on a modest machine with CPU 13th Gen Intel(R) Core(TM) i7-1355U (1.70 GHz) and RAM 16GB.

Fig. 12 ▸ shows the average running times versus the number *Z* of molecular components in a unit cell. This number *Z* goes up to 36 and coincides with *g*, because all finally optimized crystals are saved in the simplest translation group *P*1.

## 5. Continuous asymmetries of all experimental crystals in the CSD

This section describes a large-scale analysis of asymmetries in the whole CSD. Each crystal is represented by a periodic set of all its atoms. We considered all periodic crystals with complete 3D geometry, no disorder and based on a chemically unique molecule. Though Definition 5[Statement definition5] can be applied to geometric blocks of different sizes, we postpone the more complicated case of co-crystals to future work.

The snapshot of the CSD on 12 November 2025 contained 1 394 755 entries, including 907 223 crystals without disorder. Among them, 69 196 crystals have asymmetric units containing *G* ≥ 2 molecules that all have the same composition, where *g* was computed by the CSD Python API as the number of components in the list crystal.asymmetric_unit_molecules. Some crystals with the highest *Z*′ values from https://zprime.co.uk/database, such as OGUROZ (*Z*′ = 56), TMESNH (*Z*′ = 32), IDOSID (*Z*′ = 24) and VIFXEQ (*Z*′ = 24), were excluded because of disorder.

**Figure 13:**
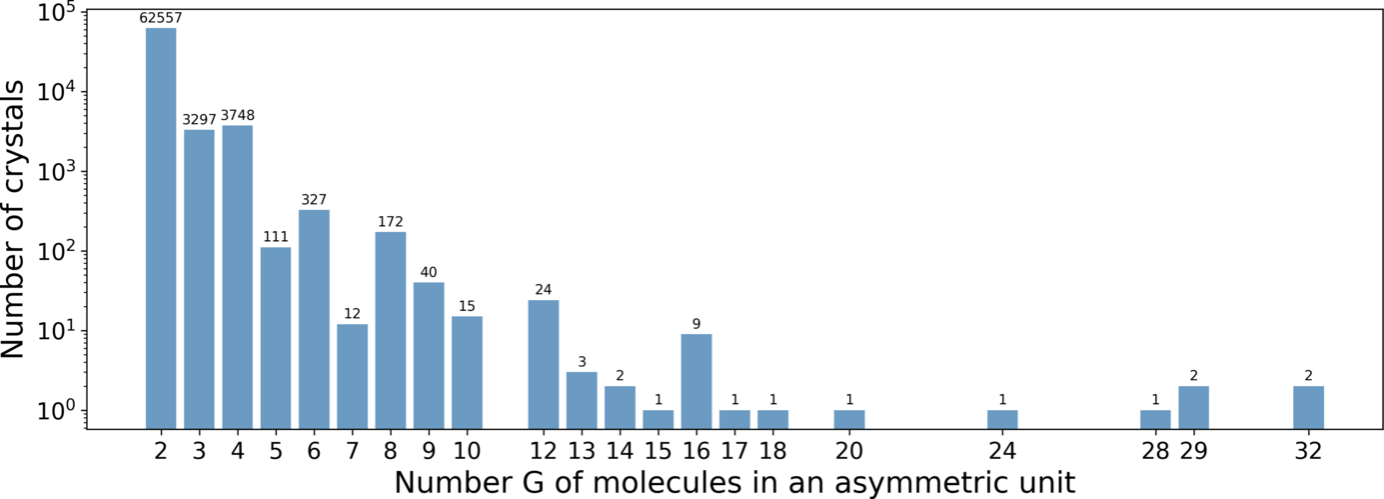
The histogram of integer numbers *g* for all 69 196 periodic crystals in the CSD that have *G* ≥ 2 chemically equivalent blocks in their asymmetric units.

Figs. 13 ▸, 14 ▸ and 15 ▸ show the histograms of *G*, *Z*′ and four CIAs for this subset of the CSD, respectively, where *Z*′ was computed as crystal.z_prime by the CSD Python API. The number *Z*[CIF] of molecules in the full motif was taken from the items _cell_formula_units_Z in CIFs from the CSD, which sometimes differ from *Z*[CSD], computed as the number of components in the list crystal.molecule.

Table 2 ▸ shows all four versions of CIAs for the most extreme crystals in the CSD: five crystals with the lowest *Z*′ ≤ 0.33 and five crystals with the highest *Z*′ ≥ 28.

**Figure 16:**
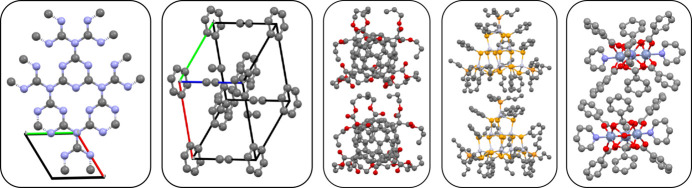
The crystals with the lowest *Z*′ from Table 2 shown without hydrogen atoms. From left to right: VESWEZ, ELIQIZ02, ZOKYEH01, RARTEK and ZAVJOV.

In Table 2 ▸, crystal VESWEZ has *g* = 2 geometric blocks CN_2_ in isometrically non-equivalent positions: in one CN_2_, both nitrogen atoms are linked to two carbon atoms; in another CN_2_, the two nitrogen atoms are linked to two and three carbon atoms, see Fig. 16 ▸. Crystal ELIQIZ02 has molecules C_6_H_6_ and C_2_H_2_, and its asymmetric unit consists of *g* = 2 isometrically different carbon atoms: one from C_6_H_6_ and another from C_2_H_2_. Crystal ZOKYEH01 consists of a big molecule of C_60_ with extra tails, but its asymmetric unit was also split into *g* = 2 blocks C_10_O_2_, which apparently have isometrically non-equivalent positions within the full crystal. Crystals RARTEK and ZAVJOV similarly consist of big molecules based on *g* = 2 blocks in asymmetric units, whose positions can not be matched by any isometry preserving the whole crystal.

**Figure 17:**
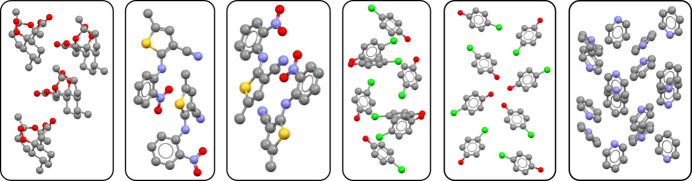
Six famous polymorphs whose CIAs are listed in Table 3. From left to right: QNGHSU01, QAXMEH31, QAXMEH57, CLPHOL12, CLPHOL13 and PYRDNA04.

Table 3  ▸includes the CIAs of the six famous polymorphs from the CSD in Fig. 17 ▸. Table 4 ▸ lists the ten crystals from the CSD with the lowest values of CIAs. The first three crystals have CIA = 0 within three decimal places, so their *Z*′ ≥ 2 might be corrected.

The value CIA = 0 means that all molecules are geometrically equivalent, *i.e.* can be exactly matched by isometry that preserves the whole crystal. In this case, an asymmetric unit should intersect only one molecule (*g* = 1), so *Z*′ ≤ 1 is expected.

**Figure 18:**
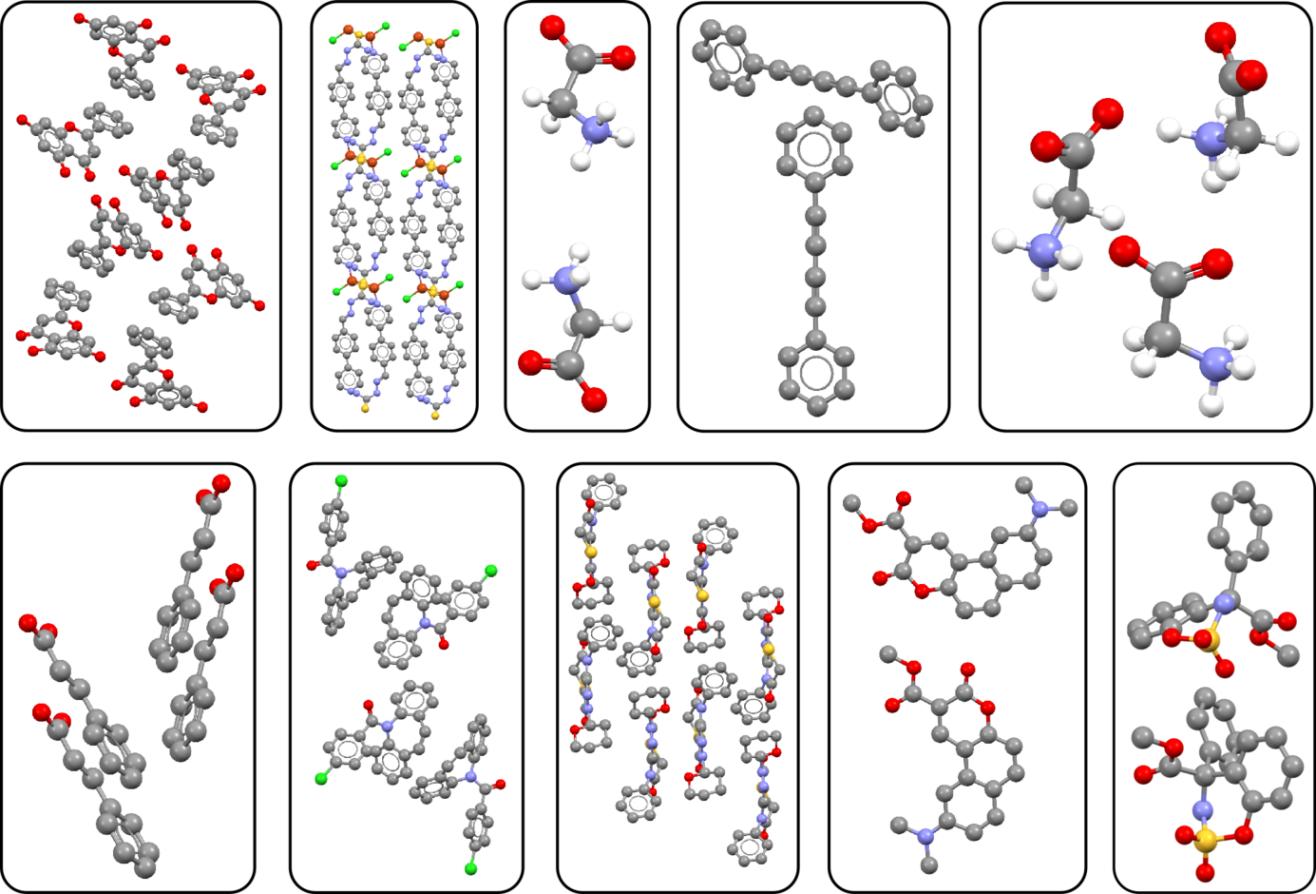
Ten crystals (some shown without hydrogen atoms) from Table 4 with very low CIA ≥ 0. Top from left to right: IYIWIY, GIBVOG, GLYCIN81, YOSNEZ05, GLYCIN82. Bottom: CINMAC13, KAVXUE, ADUWED, XOTRAB, COTZES.

**Figure 20:**
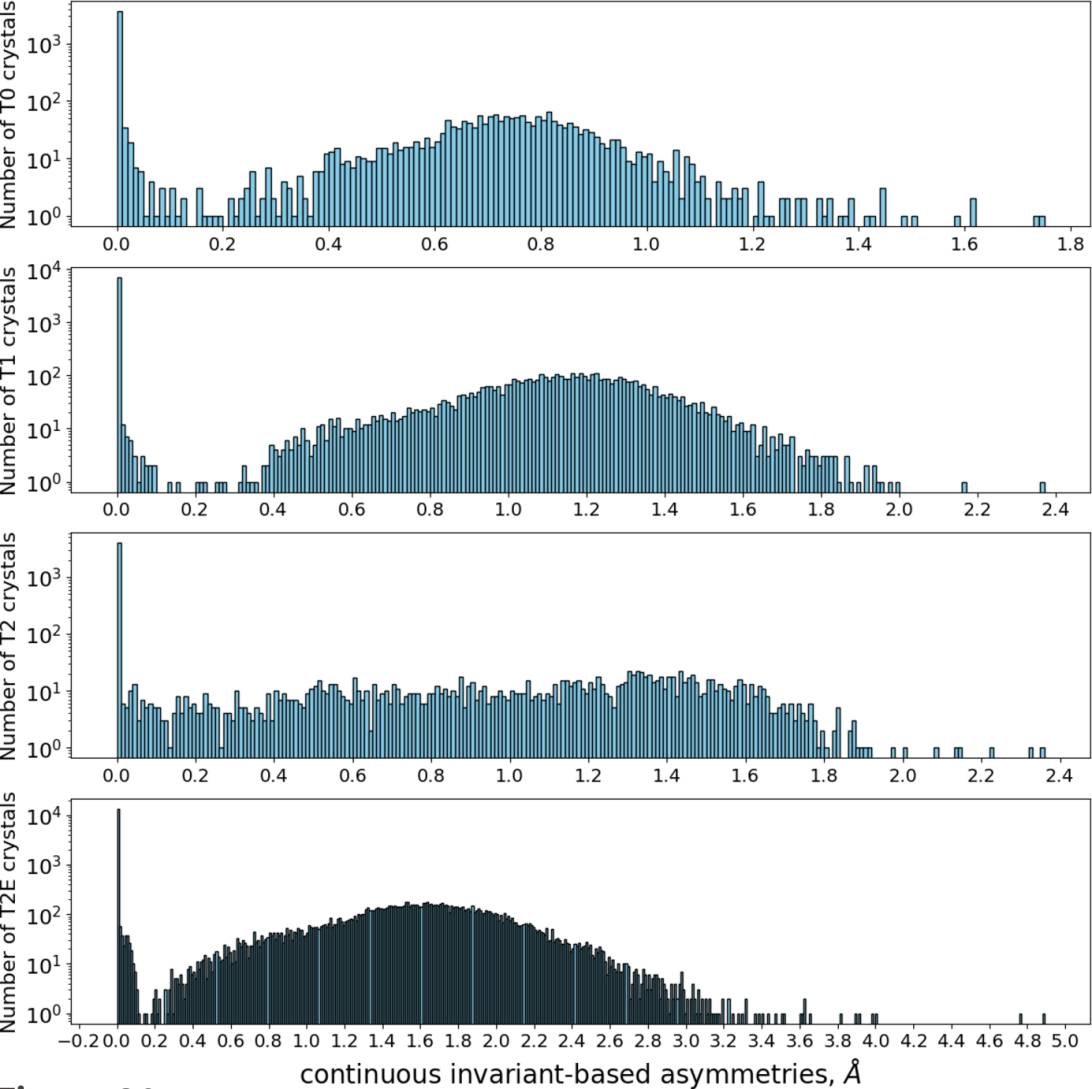
The histograms of CIA_∞_ for simulated crystals represented by three base points at ‘ends’ of molecules in Fig. 2. Row 1: T0. Row 2: T1. Row 3: T2. Row 4: T2E.

The unexpected values *Z*′ > 1 are likely explained by a wrong space group (Henling & Marsh, 2014 ▸). Crystal IYIWIY has the group *P*1, but looks more symmetric in the first picture of Fig. 18 ▸. Both structures IYIWIY and YOSNEZ05 were obtained from powder data, so their space groups might need re-checking. Since all CIAs continuously change under perturbations by Theorem 9[Statement theorem9], there is no need to search for a higher-symmetry group, which drops to the simplest group *P*1 under almost any noise.

The values of *Z*[CIF] can be corrected for all entries with *Z* < *g* in Tables 4 ▸ and 5 ▸, because a unit cell should not have fewer molecules than blocks in an asymmetric unit.

In conclusion, the relative multiplicity *Z*′ discontinuously changes under almost any perturbation, while the proposed CIA in Definition 5[Statement definition5] is continuous by Theorem 9[Statement theorem9]. For the CSP datasets in Section 4, about a half of all the over fifty thousand simulated crystals have CIA > 0, while all experimental crystals have CIA = 0, see Table 1 ▸. Moreover, these continuous and fast asymmetries are not correlated with density and energy. The large-scale experiments on the CSD show that many non-symmetric crystals with high *Z*′ have low CIAs in Table 5 ▸ and hence are geometrically close to more symmetric forms.

Further continuous asymmetries based on other invariants of periodic point sets (Anosova & Kurlin, 2021 ▸; Widdowson *et al.*, 2022 ▸; Anosova & Kurlin, 2022 ▸; Anosova & Kurlin, 2023 ▸; Kurlin, 2025 ▸) are postponed for future work.

## Figures and Tables

**Figure 5:**
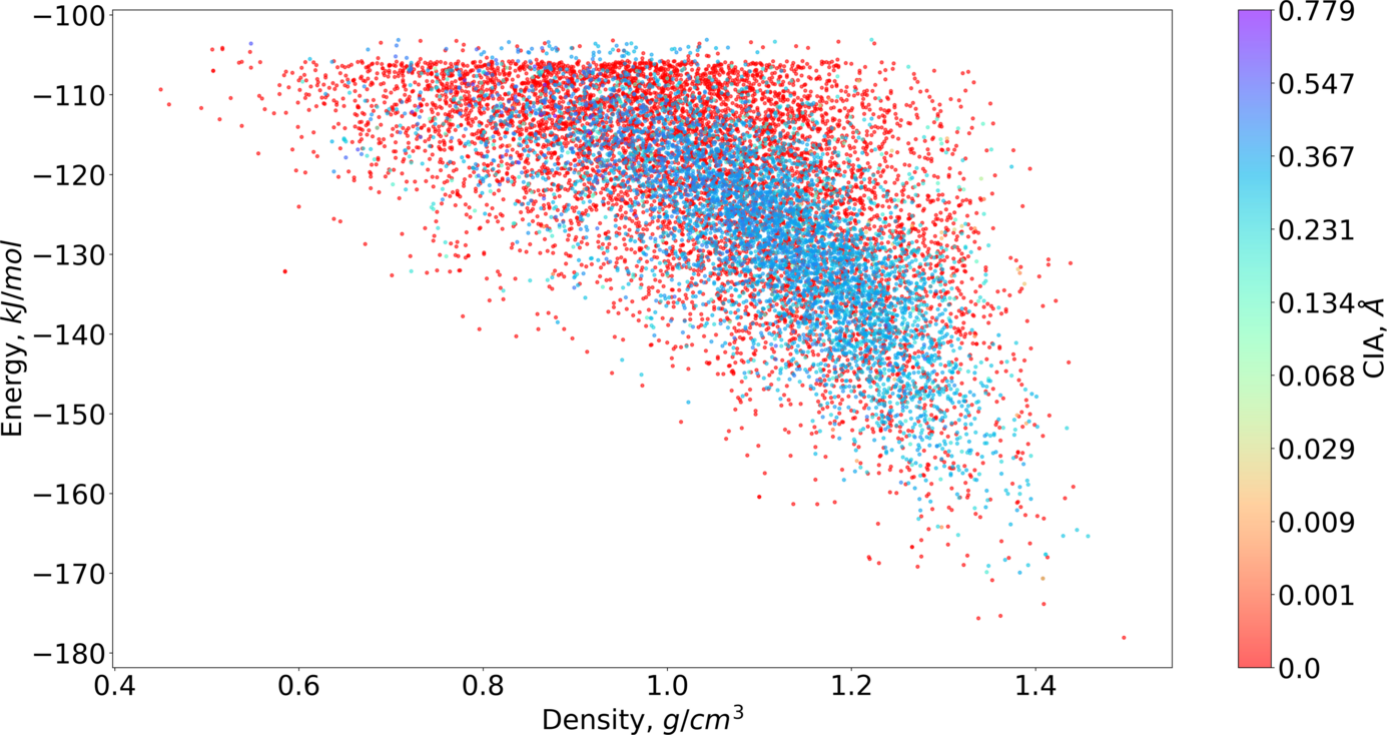
Energy versus density for simulated T1 crystals, coloured by their CIA.

**Figure 6:**
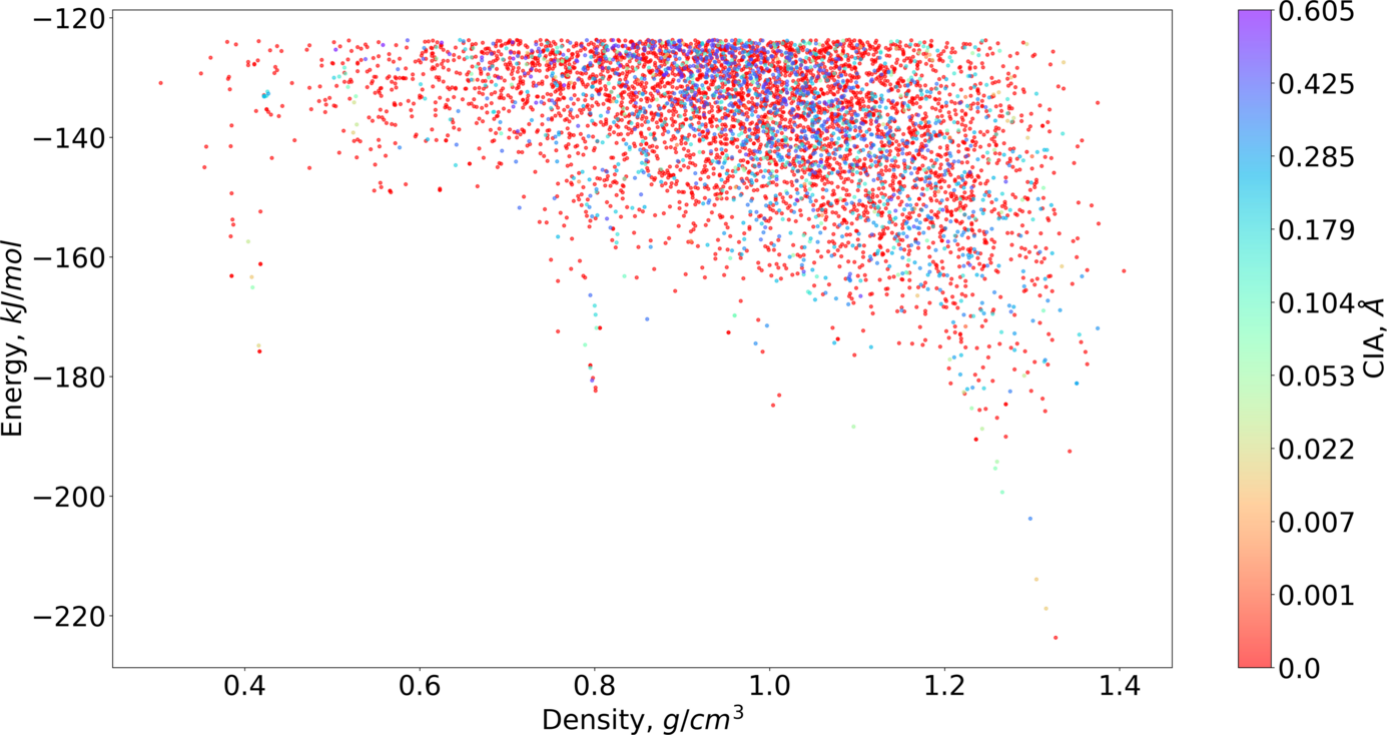
Energy versus density for simulated T2 crystals, coloured by their CIA.

**Figure 7:**
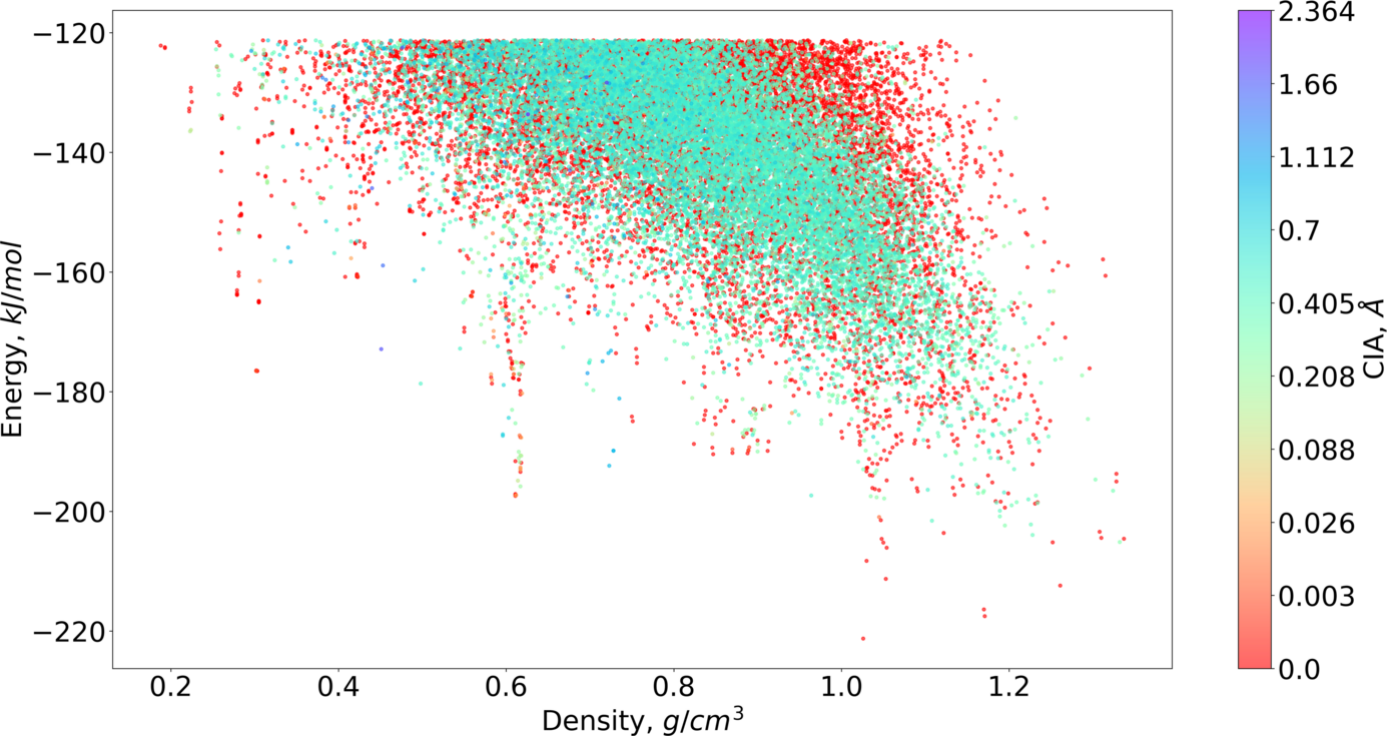
Energy versus density for simulated T2E crystals, coloured by their CIA.

**Figure 9:**
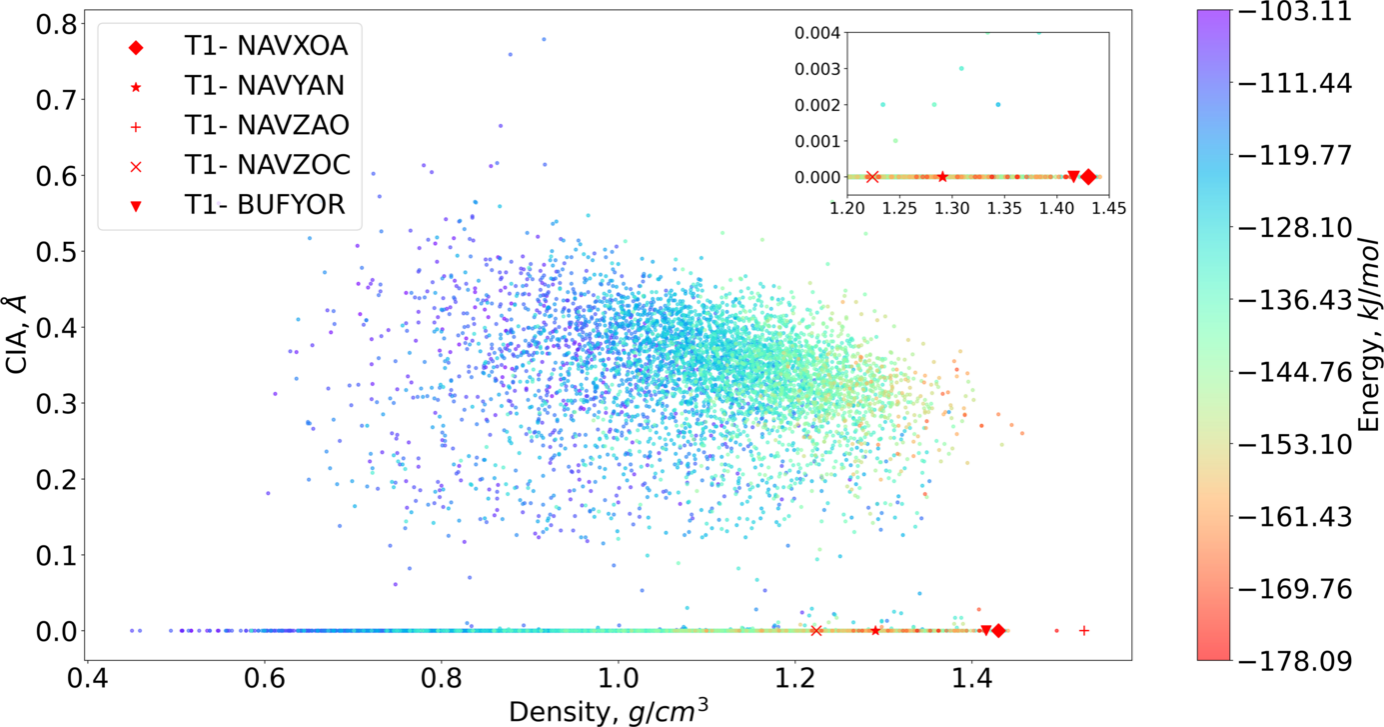
CIA versus density for simulated and experimental T1 crystals in the CSD.

**Figure 10:**
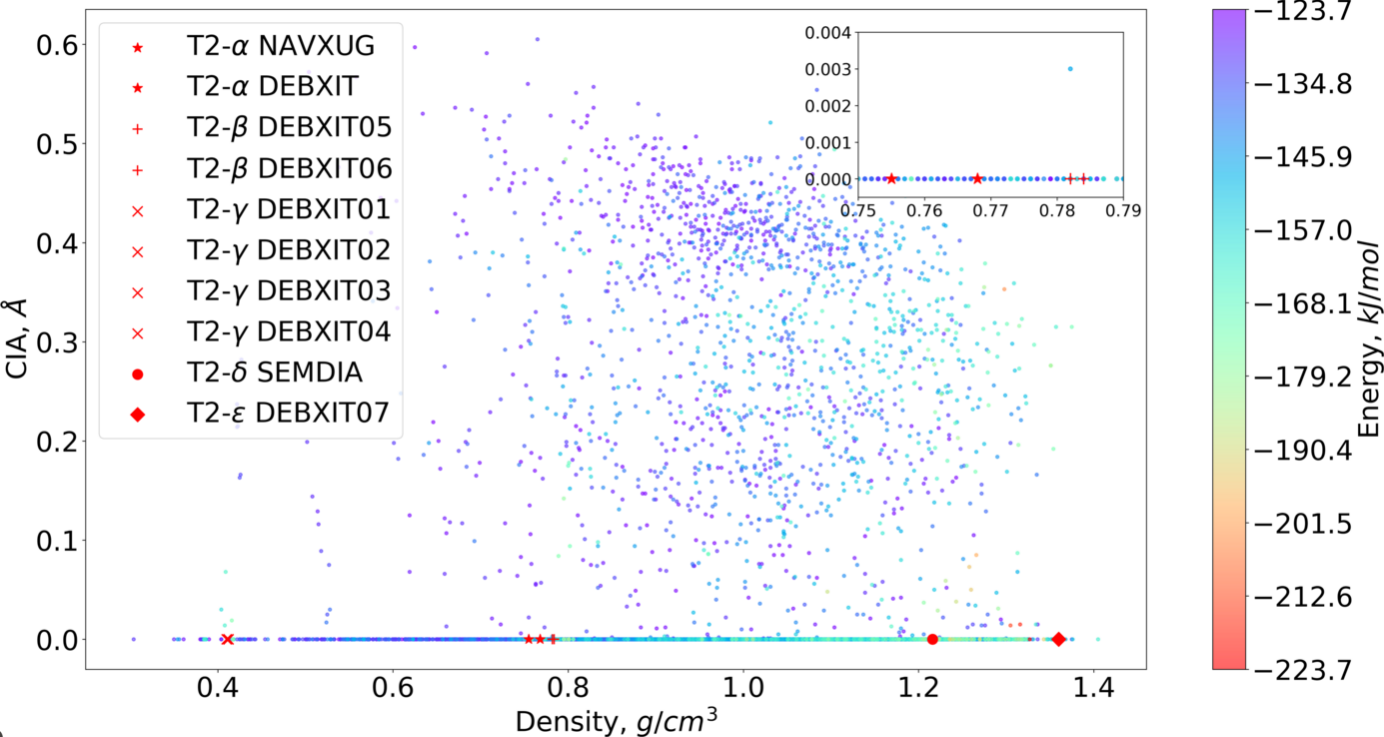
CIA versus density for simulated and experimental T2 crystals in the CSD.

**Figure 11:**
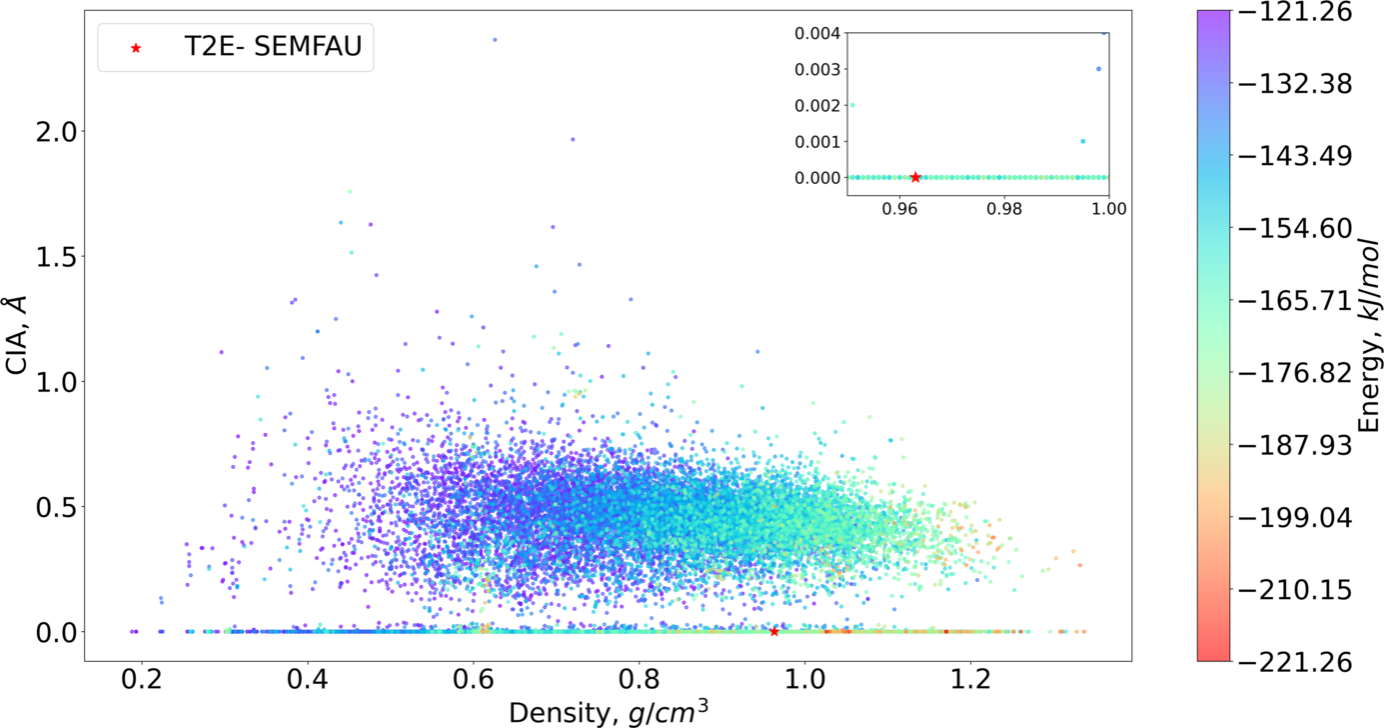
CIA versus density for simulated and experimental T2E crystals in the CSD.

**Figure 14:**
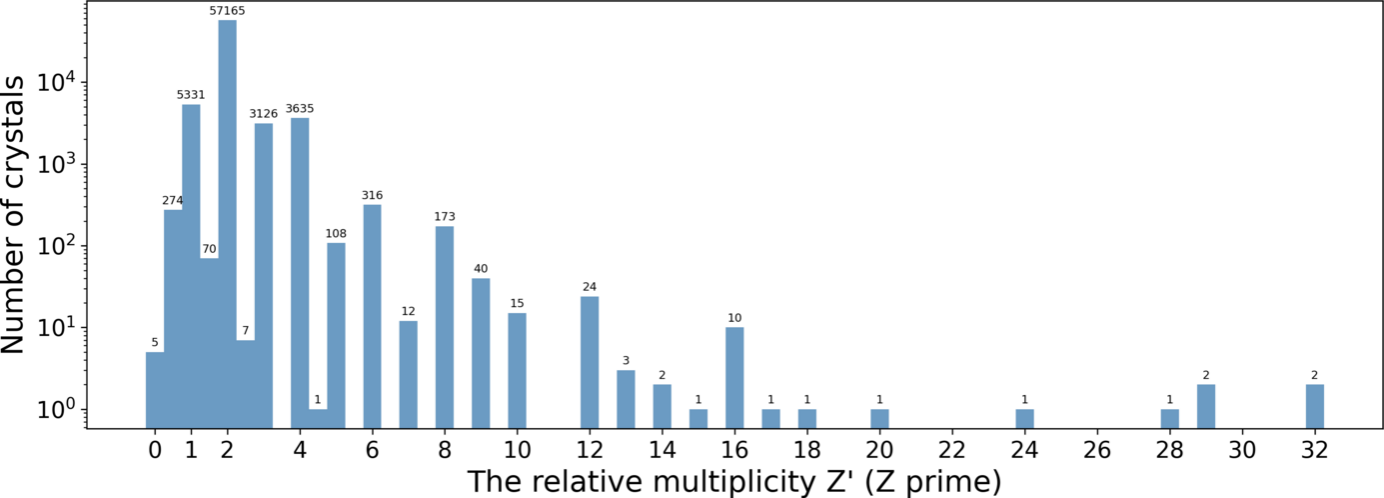
The histogram of *Z*′ with bin size 0.5 for all 69 196 periodic crystals in the CSD that have *G* ≥ 2 chemically equivalent blocks in their asymmetric units.

**Figure 15:**
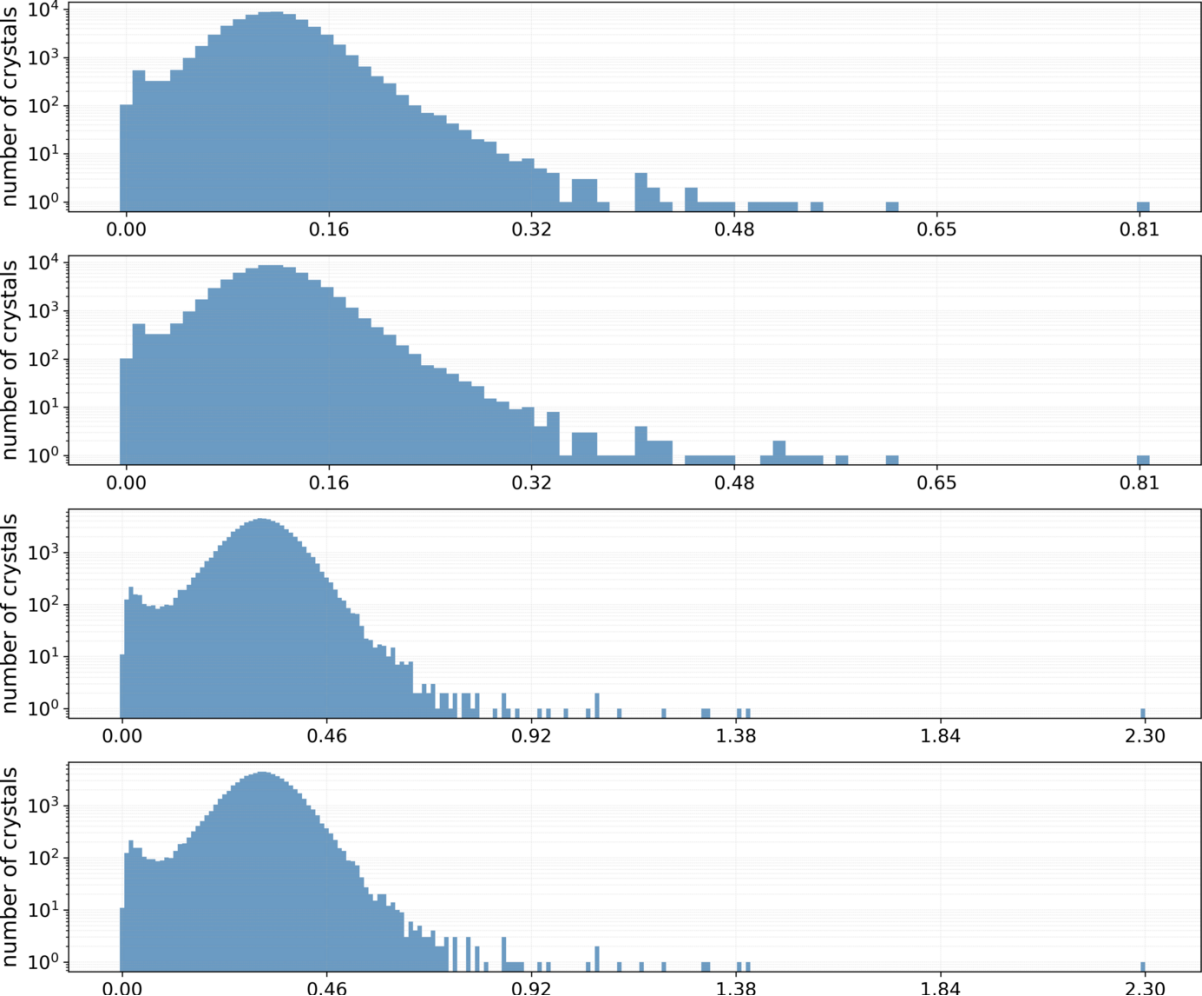
The histograms of CIAs on the log scale with bin size 0.01 Å for all 69 196 periodic crystals in the CSD that have *G* ≥ 2 chemically equivalent molecules in their asymmetric units. Row 1: CIA. Row 2: 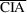. Row 3: CIA_∞_. Row 4: 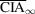.

**Figure 19:**
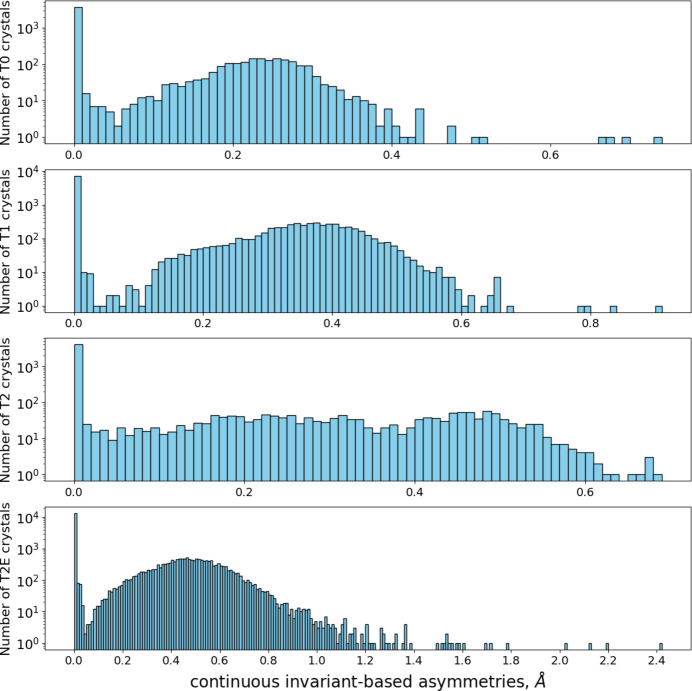
The histograms of 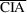 for simulated crystals represented by three base points at ‘ends’ of molecules in Fig. 2. Row 1: T0. Row 2: T1. Row 3: T2. Row 4: T2E.

**Figure 21:**
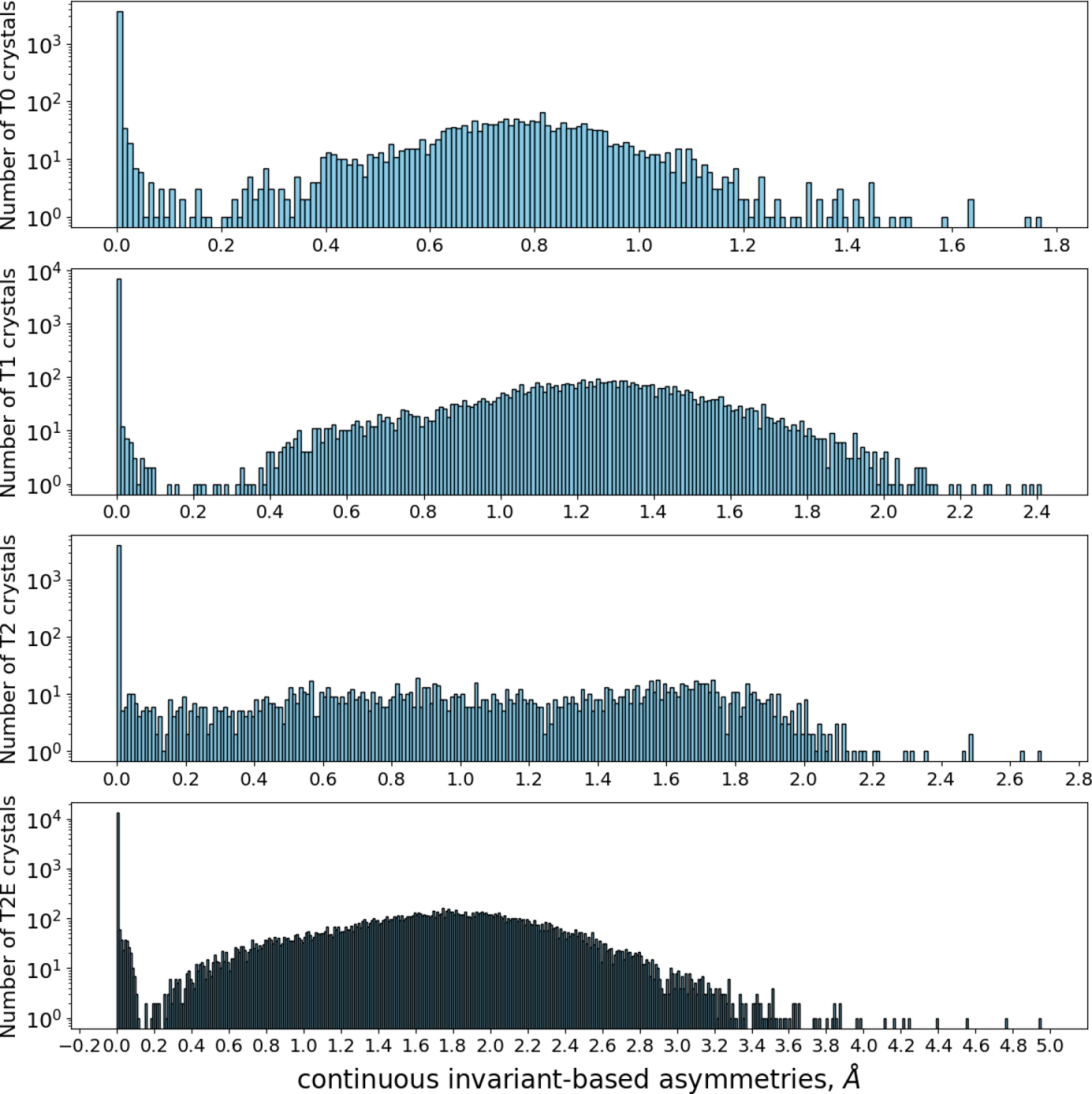
The histograms of 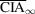 for simulated crystals represented by three base points at ‘ends’ of molecules in Fig. 2. Row 1: T0. Row 2: T1. Row 3: T2. Row 4: T2E.

**Figure 22:**
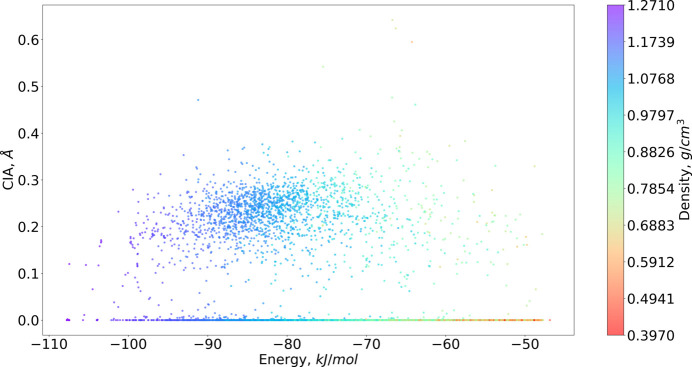
CIA versus energy plot for simulated T0 crystals, coloured by their density.

**Figure 23:**
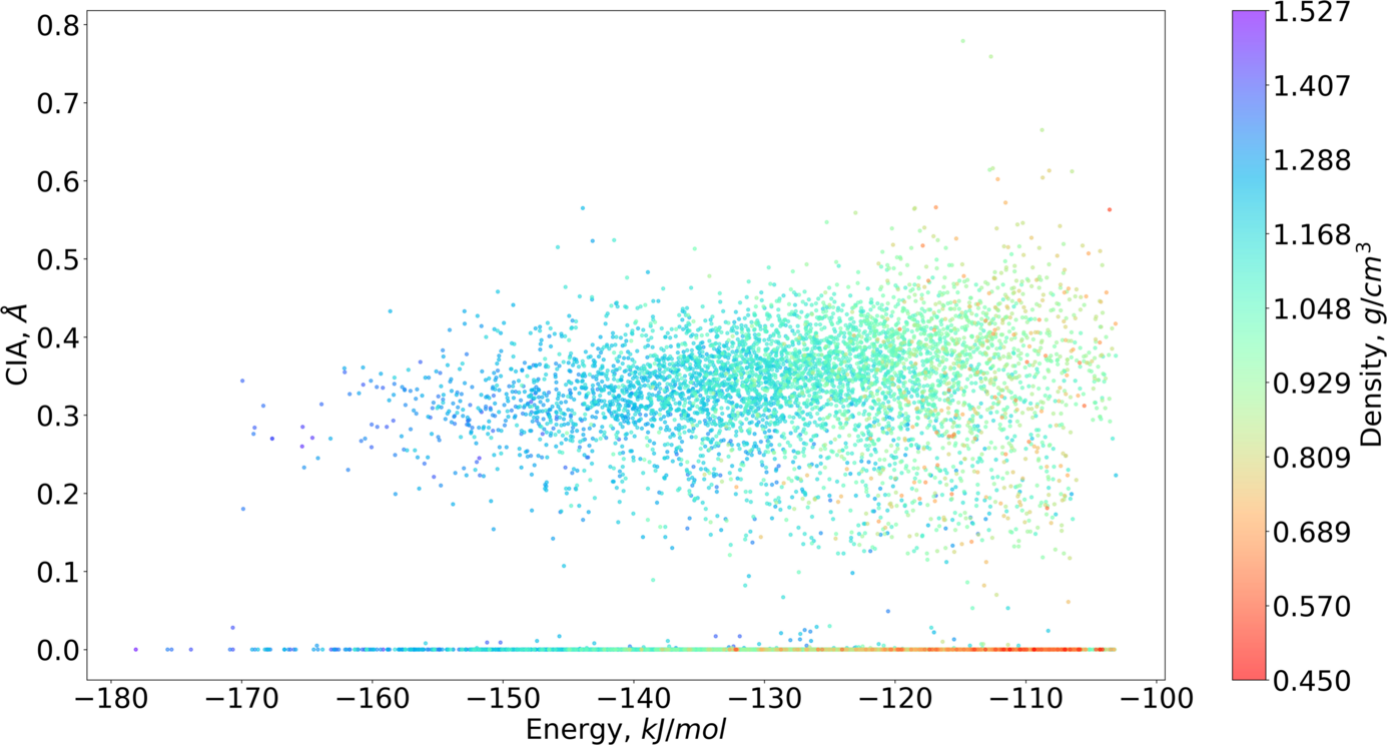
CIA versus energy plot for simulated T1 crystals, coloured by their density.

**Figure 24:**
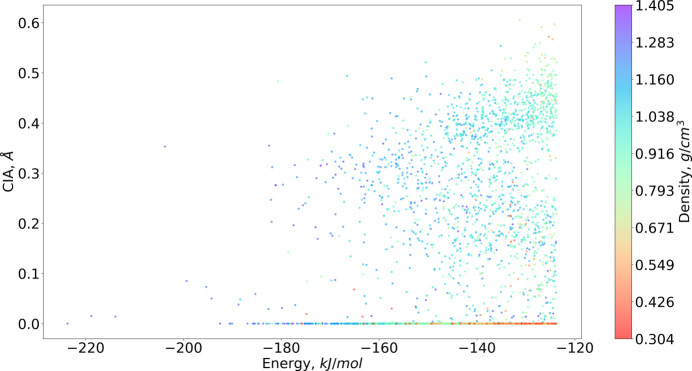
CIA versus energy plot for simulated T2 crystals, coloured by their density.

**Figure 25:**
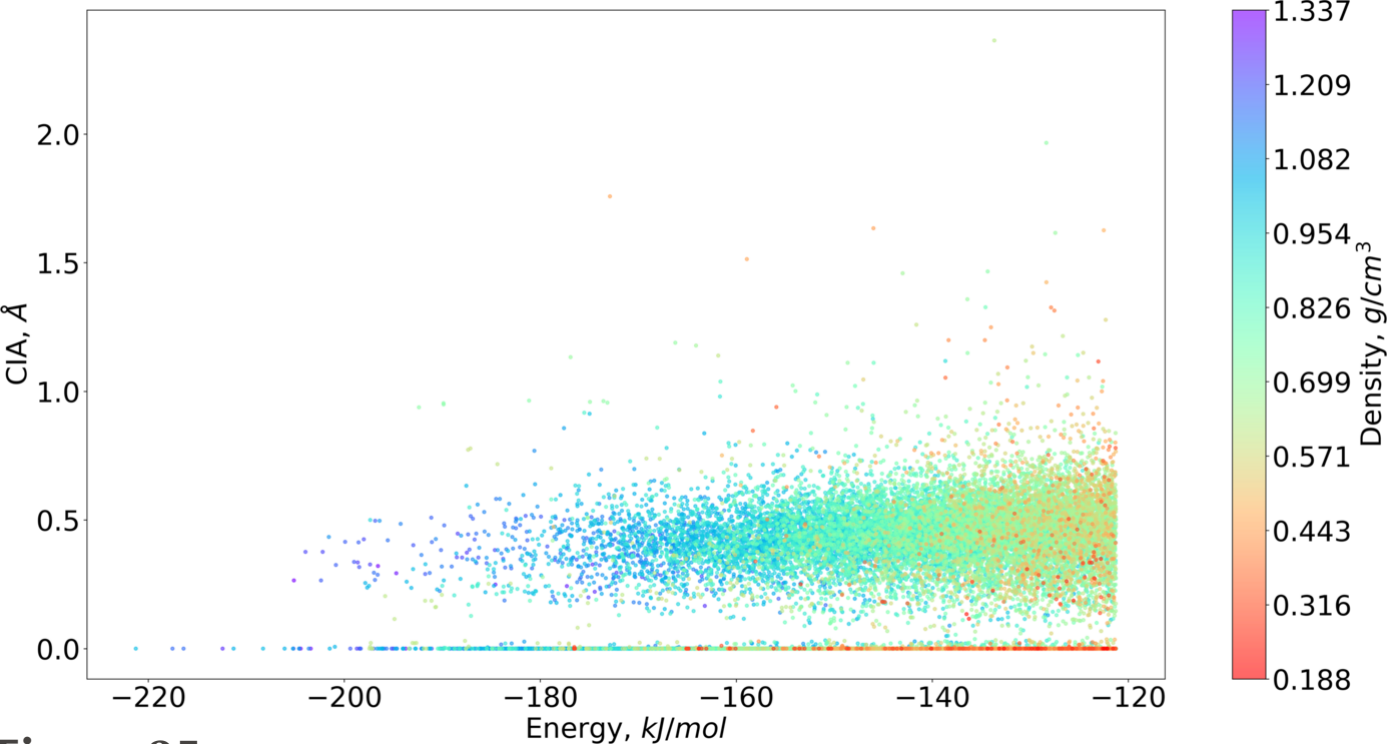
CIA versus energy plot for simulated T2E crystals, coloured by their density.

**Table 1:** Statistics of CIA values for the four CSP datasets from Pulido *et al.* (2017 ▸) The last rows contain Pearson correlations *r*(*x*, *y*) between energy, density, and new CIAs.

	CSP dataset			
	T0	T1	T2	T2E
No. of all crystals	5645	12524	5679	29908
No. of crystals: CIA ≥ 0.001	2024 ▸	5363	1687	16491
Percentage: CIA ≥ 0.001	35.8%	42.8%	29.7%	55.1%
Maximum CIA, Å	0.642	0.779	0.605	2.364
				
Correlation(energy, density)	−0.909	−0.639	−0.377	−0.500
Correlation(energy, CIA)	−0.394	−0.202	+0.022	−0.026
Correlation(density, CIA)	+0.317	+0.148	+0.040	−0.021

**Table 2:** CIAs of the crystals with the lowest and largest relative multiplicities in the CSD The numbers *Z* and *g* count molecules in a unit cell and an asymmetric unit, respectively.

CSD refcode	*Z*[CIF]	*g* blocks	*Z*′[CSD]	CIA (Å)	CIA (Å)	CIA_∞_ (Å)	CIA_∞_ (Å)
VESWEZ	2	2	0.083	0.204	0.204	0.580	0.580
ELIQIZ02	3	2	0.083	0.226	0.226	0.807	0.807
ZOKYEH01	16	2	0.167	0.086	0.086	0.241	0.241
RARTEK	16	2	0.17	0.125	0.125	0.332	0.332
ZAVJOV	2	2	0.33	0.090	0.090	0.241	0.241
							
QILJII01	112	28	28	0.168	0.185	0.397	0.426
LOFRAD	116	29	29	0.149	0.183	0.420	0.499
LOFRAD01	116	29	29	0.149	0.185	0.434	0.506
JIPTIL09	32	32	32	0.104	0.109	0.266	0.282
JIPTIL10	32	32	32	0.102	0.109	0.265	0.282

**Table 3:** CIAs of the well known polymorphs of artemisinin (QNGHSU01), pyridine (PYRDNA04), *para*-chlorophenol (α form CLPHOL12 and β form CLPHOL13), and the famous ROY molecule (R05 polymorph QAXMEH31 and R18 polymorph QAXMEH57)

CSD refcode	*Z*[CIF]	*g* blocks	*Z*′[CSD]	CIA (Å)	CIA (Å)	CIA_∞_ (Å)	CIA_∞_ (Å)
QNGHSU01	4	4	4	0.357	0.379	1.093	1.096
QAXMEH31	2	2	2	0.440	0.440	1.098	1.098
QAXMEH57	2	2	2	0.807	0.807	1.602	1.602
CLPHOL12	2	2	2	0.790	0.790	2.594	2.594
CLPHOL13	2	2	2	0.575	0.575	1.132	1.132
PYRDNA04	4	4	4	1.971	2.096	2.756	2.861

**Table 4:** Ten crystals with the lowest CIA among 69 196 periodic crystals in the CSD that have *G* ≥ *Z*′ ≥ 2 chemically equivalent blocks in their asymmetric units

CSD refcode	*Z*[CIF]	*g* blocks	*Z*′[CSD]	CIA (Å)	CIA (Å)	CIA_∞_ (Å)	CIA_∞_ (Å)
IYIWIY	8	8	8	0.000	0.000	0.000	0.000
GLYCIN81	2	2	2	0.000	0.000	0.000	0.000
YOSNEZ05	2	2	2	0.000	0.000	0.000	0.000
GIBVOG	2	2	2	0.000	0.000	0.001	0.001
GLYCIN82	3	3	3	0.001	0.001	0.002	0.002
KAVXUE	1	2	2	0.002	0.002	0.005	0.005
ADUWED	64	2	2	0.002	0.002	0.006	0.006
CINMAC13	2	2	2	0.002	0.002	0.010	0.010
XOTRAB	4	2	2	0.003	0.003	0.007	0.007
COTZES	6	2	2	0.003	0.003	0.009	0.009

**Table 5:** Almost symmetric crystals with high values *Z*′ ≥ 5 but low CIA ≤ 0.021 Å

CSD refcode	*Z*[CIF]	*g* blocks	*Z*′[CSD]	CIA (Å)	CIA (Å)	CIA_∞_ (Å)	CIA_∞_ (Å)
TEGBEP	1	6	6	0.010	0.011	0.030	0.032
HOGKAR	12	6	6	0.010	0.011	0.032	0.034
GINHIX	6	6	6	0.011	0.012	0.034	0.039
EVIWUE	12	6	6	0.012	0.014	0.051	0.059
LEMWOR	2	6	6	0.013	0.013	0.040	0.043
YIVHER	10	5	5	0.015	0.016	0.053	0.058
IFOFAN	10	5	5	0.020	0.020	0.070	0.077
EDUCAL	12	6	6	0.020	0.022	0.062	0.071
ROTSAY	18	9	9	0.021	0.023	0.060	0.067
CIDHAB	1	12	12	0.021	0.023	0.067	0.071

**Table 6:** Statistics of 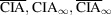 for the four CSP datasets from Pulido *et al.* (2017 ▸) The last rows contain Pearson correlations *r*(*x*, *y*) between energy, density, and new CIAs.

	CSP dataset			
	T0	T1	T2	T2E
maximum CIA_∞_, Å	1.748	0.902	2.352	4.882
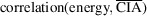	−0.393	−0.196	+0.035	−0.020
correlation(energy, CIA_∞_)	−0.398	−0.196	+0.016	−0.019
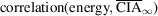	−0.399	−0.186	+0.032	−0.014
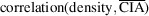	+0.315	+0.144	+0.036	−0.021
correlation(density, CIA_∞_)	+0.322	+0.133	+0.037	−0.033
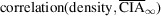	+0.323	+0.131	+0.032	−0.022

## Appendix A Extra experimental results for simulated crystals

This appendix illustrates three other versions of CIAs from Definition 5[Statement definition5] in addition to the default Continuous Invariant-based Asymmetry (CIA). For example, Table 6 ▸ and Figs. 19 ▸, 20 ▸ and 21 ▸ illustrate ranges of CIAs similar to Table 1 ▸ and Fig. 3 ▸. Figs. 22 ▸, 23 ▸, 24 ▸ and 25 ▸ plot the CIA (Å) versus the (lattice) energy (kJ mol^−1^) for T0, T1, T2 and T2E simulated crystals, respectively.

The colour bar for density is shown at the right side of each plot. While symmetric crystals exist across the full range of energies and densities, crystals with higher energies tend to have larger asymmetries. High-density structures can have both zero and non-zero asymmetries, indicating that density alone does not determine symmetry or thermodynamic stability.

## Appendix B Detailed proofs of mathematical results

Proof of Lemma 7 Let an asymmetric unit *A* of a periodic point set  be replaced with another asymmetric unit *A*′ such that *S* ∩ *A* and *S* ∩ *A*′ have the same size. Since both *A*, *A*′ can be expanded by symmetry operations of *S* to the same periodic set *S*, there is a bijection β : *S* ∩ *A* → *S* ∩ *A*′ between finite sets of points that respects their global neighbourhoods. In other words, for any point *p* ∈ *S* ∩ *A*, there is an isometry *f* of  such that *f*(*p*) = β(*p*) and *f*(*S*) = *S*. This β induces a bijection between geometric blocks if the splitting into blocks is fixed in advance, *e.g.* into connected parts of molecules as in CIFs of periodic crystals. Since the bijection is realized by global isometries of , which preserve all inter-point distances, the corresponding geometric blocks of *A*, *A*′ have the same distributions of rows in PDA(*S*; *k*) from Definition 5[Statement definition5]. Then all distances EMD(*B*_*i*_, *B*_*j*_) and *d*_*i*_, and hence CIA(*S*) in Definition 5[Statement definition5], have the same values for both *A*, *A*′. As a partial case, if an asymmetric unit *A* is transformed by a matrix from the group  or by any isometry of , all inter-point distances remain the same same, and hence CIA(*S*) is preserved. Let asymmetric units *A*, *A*′ differ by sizes. Each of them can be expanded to a primitive unit cell of *S* that has a minimum number of points. We now check that scaling *A* by any integer factor *c* > 1 keeps CIA(*S*). The set *S* ∩ *A* transforms into the *c*-times larger set containing *c* isometric copies of *S* ∩ *A*. The scaled-up asymmetric unit *A*′ contains *c* times more blocks , which can be considered *c* copies of the original blocks. The matrix of pairwise distances EMD(*B*_*i*_, *B*_*j*_) between *cg* blocks consists of *c* × *c* copies of the original matrix *g* × *g* of EMD values. The distances to the farthest units  are obtained by concatenating *c* copies of the original vector (*d*_*i*1_, …, *d*_*ig*_). Then the maximum and average values for each vector remain the same, so CIA(*S*) remains invariant under scaling of an asymmetric unit. All arguments given above similarly apply to all other versions of CIA. □

Proof of Lemma 8 The inequalities CIA(*S*) ≤ CIA_∞_(*S*) and  hold, because the RMS distance *d* is bounded from above by the Chebyshev distance *d*_∞_. The inequality  holds, because  = . To prove the inequality , let *B*_*i*_minimize  = . For *j*, *k* = 1, …, *g*, the triangle inequality

implies that for each *k* = 1, …, *g*. Then . □

Proof of Theorem 9 By Lemma 4.1 in Edelsbrunner *et al.* (2021 ▸), if periodic point sets  are related by an ɛ-perturbation with ɛ < *r*(*S*), then *S*, *Q* have a common lattice. Since CIA is invariant under changes of a unit cell by Lemma 7[Statement lemma7], we can assume that *S*, *Q* have the same number *m* of points in a common unit cell and equal Point Packing Coefficients PPC(*S*) = PPC(*Q*) in Definition 3[Statement definition3]. By Lemma SM3.4 in Widdowson & Kurlin (2026 ▸), all corresponding elements of PDD(*S*; *k*), PDD(*Q*; *k*) differ by at most 2ɛ, which generalizes the fact that perturbing any two points up to ɛ changes the distance between them up to 2ɛ by the triangle inequality. The same upper bound of 2ɛ holds for differences between all corresponding elements of PDA(*S*; *k*), PDA(*Q*; *k*) in Definition 3[Statement definition3] due to PPC(*S*) = PPC(*Q*). For both Chebyshev and Root Mean Square distances between rows of *k* distances, the upper bound of 2ɛ between corresponding distances |*b*_*i*_ − *c*_*i*_| ≤ 2ɛ, *i* = 1…, *k*, guarantees the same upper bound for and

Let *B*_1_, …, *B*_*g*_ be all geometric blocks in an asymmetric unit of *S*. Denote by *C*_1_, …, *C*_*g*_ the corresponding blocks in an asymmetric unit of *Q* so that each *C*_*i*_ is an ɛ-perturbation of *B*_*i*_ for *i* = 1, …, *g*. By the argument above, all *m*_*i*_ corresponding points of *B*_*i*_ and *C*_*i*_ have 2ɛ-close rows in PDA(*S*; *k*) and PDA(*Q*; *k*), respectively, for *i* = 1, …, *g*. Then *d*(*R*_*j*_(*B*_*i*_), *R*_*j*_(*C*_*i*_)) ≤ 2ɛ for *j* = 1, …, *m*_*i*_, where the ground distance *d* is Chebyshev or RMS. In the notations of Definition 4[Statement definition4], if we set  for *j* = 1, …, *m*_*i*_, else 0, then EMD(*B*_*i*_, *C*_*i*_) ≤ 2ɛ. The triangle inequality implies that

Swapping the *B*-blocks and *C*-blocks, we similarly get EMD(*C*_*i*_, *C*_*j*_) ≤ EMD(*B*_*i*_, *B*_*j*_) + 4ɛ and |EMD(*B*_*i*_, *B*_*j*_) − EMD(*C*_*i*_, *C*_*j*_)| ≤ 4ɛ, so the corresponding elements of the matrix of EMDs differ by at most 4ɛ. Then the maximum distances *d*_*i*_ in Definition 5[Statement definition5] and hence the minima and averages over *j* = 1, …, *g* differ by at most 4ɛ. □

Proof of Theorem 10 By Definition 5[Statement definition5], starting with the matrix PDA(*S*; *k*), we need *O*(*g*^2^) distances EMD(*B*_*i*_, *B*_*j*_) for all 1 ≤ *i* < *j* ≤ *g*. Every Earth Mover’s Distance EMD(*B*_*i*_, *B*_*j*_) is exactly computed in time  for any distributions of a maximum size *m* (Orlin & Ahuja, 1992 ▸). Then all CIAs are computed in extra time *O*(*g*^2^). For the splitting into *m* single-point blocks, every distance EMD(*p*_*i*_, *p*_*j*_) is RMS and *L*_∞_ between rows of *m* values, which requires time *O*(*m*). The total time to get each CIA in Definition 5[Statement definition5] from *O*(*m*^2^) distances EMD(*p*_*i*_, *p*_*j*_) is *O*(*m*^3^). □
